# EFR3A: a new raft domain organizing protein?

**DOI:** 10.1186/s11658-023-00497-y

**Published:** 2023-10-25

**Authors:** Magdalena Trybus, Anita Hryniewicz-Jankowska, Karolina Wójtowicz, Tomasz Trombik, Aleksander Czogalla, Aleksander F. Sikorski

**Affiliations:** 1 Department of Cytobiochemistry, Faculty of Biotechnology, University of Wroclaw, F. Joliot-Curie 14a, 50-383 Wroclaw, Poland; 2 Department of Biotransformation, Faculty of Biotechnology, University of Wroclaw, F. Joliot-Curie 14a, 50-383 Wroclaw, Poland; 3 Chair and Department of Biochemistry and Molecular Biology, Medical University of Lublin, Chodzki 1, 20-093 Lublin, Poland; 4 Research and Development Center, Regional Specialist Hospital, Kamienskiego73a, 51-154 Wroclaw, Poland

**Keywords:** Raft domain organization and regulation, Flotillins, Flotillin-2, EFR3A, Membrane order, FLIM, svFCS, EGFR

## Abstract

**Background:**

Membrane rafts play a crucial role in the regulation of many important biological processes. Our previous data suggest that specific interactions of flotillins with MPP1 are responsible for membrane raft domain organization and regulation in erythroid cells. Interaction of the flotillin-based protein network with specific membrane components underlies the mechanism of raft domain formation and regulation, including in cells with low expression of MPP1.

**Methods:**

We sought to identify other flotillin partners via the immobilized recombinant flotillin-2-based affinity approach and mass spectrometry technique. The results were further confirmed via immunoblotting and via co-immunoprecipitation. In order to study the effect of the candidate protein on the physicochemical properties of the plasma membrane, the gene was knocked down via siRNA, and fluorescence lifetime imaging microscopy and spot-variation fluorescence correlation spectroscopy was employed.

**Results:**

EFR3A was identified as a candidate protein that interacts with flotillin-2. Moreover, this newly discovered interaction was demonstrated via overlay assay using recombinant EFR3A and flotillin-2. EFR3A is a stable component of the detergent-resistant membrane fraction of HeLa cells, and its presence was sensitive to the removal of cholesterol. While silencing the *EFR3A* gene, we observed decreased order of the plasma membrane of living cells or giant plasma membrane vesicles derived from knocked down cells and altered mobility of the raft probe, as indicated via fluorescence lifetime imaging microscopy and spot-variation fluorescence correlation spectroscopy. Moreover, silencing of *EFR3A* expression was found to disturb epidermal growth factor receptor and phospholipase C gamma phosphorylation and affect epidermal growth factor-dependent cytosolic Ca^2+^ concentration.

**Conclusions:**

Altogether, our results suggest hitherto unreported flotillin-2-EFR3A interaction, which might be responsible for membrane raft organization and regulation. This implies participation of this interaction in the regulation of multiple cellular processes, including those connected with cell signaling which points to the possible role in human health, in particular human cancer biology.

**Supplementary Information:**

The online version contains supplementary material available at 10.1186/s11658-023-00497-y.

## Background

The raft hypothesis, which was formulated by Simons and Ikonen in 1997 [1] and is being continuously refined by many researchers, formalizes the type of lateral heterogeneity of the cell membranes (see reviews [2–4]). It is based on preferential lateral associations of cholesterol with certain types of membrane phospholipids, among which sphingolipids play a dominant role. While the molecular mechanism of lateral phase separation in the model membrane systems seems relatively well understood, limited data are available on the biological mechanism(s) controlling the formation of raft domains in natural membranes of living cells (reviewed in [5]).

There is a reasonable agreement among researchers that relatively unstable nanoscale raft precursors within the membrane, namely, mostly oligomeric protein–lipid complexes, may become functional while associating into larger (~ ≥ 20 nm in diameter), more stable (*τ*_1/2_ > 1 s), and possibly detergent-resistant nanodomains, also known as membrane/lipid rafts and more precisely, resting-state rafts. Further clustering of the latter leads to the formation of micrometer scale raft platforms, e.g., immunological synapses [6, 7].

The list of physiological functions of rafts includes several important biological processes, such as cellular signal transduction, immune signaling involving several innate and adaptative immune responses [8, 9], host–pathogen interactions, e.g., severe acute respiratory syndrome coronavirus 2 (SARS-CoV-2) [10], cancerogenesis [11, 12] and other pathologies, e.g., atherosclerosis (reviewed e.g., by Kwiatkowska et al. [13]).

Membrane proteins of the SPFH family (stomatin/prohibitin/flotillin/HflK) sharing common SPFH domains, such as flotillin-1 and -2 and stomatin or stomatin-like proteins, are characteristic features of the raft domains [14].

Taking into account the physiological role of raft domains an important question is how the raft domains are formed and regulated. It should be noted that it predominantly concerns the organization of domains corresponding to resting state rafts as defined above. Hypotheses on membrane raft organization and regulation (reviewed by Sezgin et al. [15]) consider a variety of intermolecular interactions, such as lipid–lipid interactions within the lipid bilayer, lipid–protein interactions, and protein–protein binding. Moreover, the actin-skeleton has been widely considered to affect lateral membrane organization due to its effect on lateral diffusion (hop and trapped diffusion) and clustering of membrane protein and lipid components [16, 17].

EFR3A is one of the two isoforms of the 821 amino acid residue (92.9 kDa) adapter protein EFR3 involved in the recruitment of PI4KA kinase to the plasma membrane [18, 19] and thus involved in phosphoinositide metabolism and signaling. In humans, it is encoded by the *EFR3A* gene located on chromosome 8. Mutations in this gene have been suggested to be related to autism [20], gastrointestinal defects, and immunodeficiency syndrome 1 [21, 22]. The yeast EFR3N, a large N-terminus (residues 9-562) is the only available structure of EFR3 proteins is in yeast, with a large N-terminus, EFR3N, and it is α-helical, with the exception of a loop region between residues 217 and 232, forming an almost straight rod 120 Å in length composed of 27 short α-helices [23]. The N-terminus (helices 1–8) forms a Vps27p, Hrs, STAM (VHS) domain, a 153 amino acid residue motif [24] that is also present in several other proteins believed to be involved in tyrosine kinase receptor signaling.

Our previous studies on erythroid cell membrane lateral organization led to the discovery of palmitoylated membrane protein 1 (MPP1) as a major factor responsible for resting-state raft organization and regulation as reflected by the high dependence of the membrane lateral organization on this protein [25] and the direct interaction of MPP1 with flotillins as revealed via cross-linking, co-immunoprecipitation (Co-IP), pull-down, and proximity assays [26]. Moreover, the interactions were characterized in greater detail by using bacterially expressed recombinant MPP1 and its fragments with recombinant flotillins-1 and -2 and surface plasmon resonance (SPR) technique [27], which revealed that this interaction is characterized by similar affinities towards both flotillins (K_D_ within nanomolar range, 23 and 31 nM for flotillin-1 and -2, respectively).

We assume that the formation of a flotillin-based protein network interacting with specific lipids underlies the mechanism of raft domain formation and regulation in cells with limited expression of *MPP1* [28]. To address this question, an immobilized recombinant flotillin-2-based affinity approach to pull-down and identify the protein partner via tandem mass spectrometry (MS) technique was utilized. An initial observation of EFR3A being a putative flotillin-2 partner was confirmed via western blotting (WB) with anti-EFR3 antibodies, co-immunoprecipitation (Co-IP) on HeLa cells, and overlay assay on purified recombinant proteins. To explore the possibility of the role of this interaction in raft domain organization, silencing of *EFR3A* gene expression in a HeLa cell line was conducted. This allowed us to perform fluorescence lifetime imaging microscopy (FLIM) and spot-variation fluorescence correlation spectroscopy (svFCS) measurements on the membrane fluidity and raft–probe mobility changes upon the decrease of EFR3A in the membrane. Finally, we tested whether silencing of this gene expression affects the epidermal growth factor receptor (EGFR) signaling pathway in HeLa cells.

## Methods

### Reagents

A list of all antibodies used in this study can be found in Table 1. Protein G Dynabeads, di-4 ANEPPDHQ (Invitrogen, D36802), Bodipy SM, propidium iodide—1.0 mg/ml, Pierce high-capacity Ni-IMAC resin, EDTA compatible, Fluo-4 Direct calcium assay kit, and Amplex Red cholesterol assay kit were from Thermo Fisher Scientific (Waltham, MA, USA). Dulbecco’s modified Eagle medium (DMEM) without L-glutamine, fetal bovine serum (FBS), glutamine GlutaMAX (100×), 100 units/ml penicillin and 100 µg/ml streptomycin, 1× phosphate-buffered saline (PBS), trypsin–EDTA 0.05%, and Hank’s balanced salt solution (HBSS) were from Gibco (Waltham, MA, USA). Puromycin was from Santa Cruz (Dallas, TX, USA). Complete protease inhibitor cocktail was from Roche (Basel, Switzerland). Radiance ECL was from Azure Biosystems (Dublin, CA, USA). Dimethyl sulfoxide; methyl sulfoxide (DMSO), ribonuclease A (RNase A) 10 mg/ml, and glycine were from VWR (Radnor, PA, USA). Nitrocellulose membranes were from Amersham, GE Healthcare Life (Chalfont St Giles, UK). Kanamycin was from Bioshop (Burlington, ON, Canada). Of the remaining reagents and materials, octyl β-d-glucopyranoside, cyanogen bromide-activated Sepharose 4B, methyl-β-cyclodextrin (MβCD), *N*-ethylmaleimide (NEM), lysozyme, imidazole, 1,4-dithiothreitol (DTT), BCA protein assay kit, and Tween 20 were from Merck (Sigma) KGaA (Darmstadt, Germany), while 2-[4-(2-hydroxyethyl)piperazin-1-yl]ethane-1-sulfonic acid (HEPES), isopropyl β-D-1-thiogalactopyranoside (IPTG), phenylmethylsulfonyl fluoride (PMSF), sodium dodecyl sulfate (SDS), Tris–HCl, Triton X-100, sucrose, and ROTIPHORESE NF-Acrylamide/Bis-solution 30 (29:1) were from Roth (Karlsruhe, Germany).

**Table 1:** Antibodies used in this project

Antibodies	Experiment
Rabbit anti-EFR3A (Abnova GmbH, Taipei City, Taiwan)	WB 1:1000
Mouse anti-flotillin-2 (Santa Cruz Biotechnology, Dallas, TX, USA)	WB 1:1000; IP: 5 μg
Mouse anti-FLAG (Merck KGaA, Darmstadt, Germany)	WB 1:1000
Rabbit anti-EGFR (Cell Signaling, Danvers, MA, USA)	WB 1:1000
Mouse anti-flotillin-1 (Santa Cruz Biotechnology, Dallas, TX, USA)	WB 1:1000
Goat anti-flotillin-2 (Abnova GmbH, Taipei City, Taiwan)	WB 1:1000; IP: 5 μg
Mouse anti-GAPDH (Santa Cruz Biotechnology, Dallas, TX, USA)	WB 1:1000
Goat IgG (Santa Cruz Biotechnology, Dallas, TX, USA)	IP: 5 μg
Mouse IgG (Santa Cruz Biotechnology, Dallas, TX, USA)	IP: 5 μg
Anti-rabbit IgG (HRP) (Jackson ImmunoResearch, Ely, Cambridgeshire, United Kingdom)	WB 1:10,000
Anti-mouse IgG (HRP) (Jackson ImmunoResearch, Ely, Cambridgeshire, United Kingdom)	WB 1:10,000
Anti-goat IgG (HRP) (Santa Cruz Biotechnology, Dallas, TX, USA)	WB 1:10,000
Rabbit polyclonal anti-phospho-PLCγ1 Y783 (Cell Signaling, Danvers, MA, USA)	WB 1:1000
Rabbit polyclonal anti-PLCγ1 (Cell Signaling, Danvers, MA, USA)	WB 1:1000
Rabbit monoclonal phospho-EGF Receptor (Tyr1068) (D7A5) (Cell Signaling, Danvers, MA, USA)	WB 1:1000
Rabbit monoclonal EGF Receptor (D38B1) (Cell Signaling, Danvers, MA, USA)	WB 1:1000

### Cell culture

The HeLa cell line used in this study was purchased from Sigma Aldrich (Saint Louis, MO, USA) and cells were cultured in DMEM medium containing 10% fetal bovine serum, 2 mM glutamine GlutaMAX, 100 units/ml penicillin, and 100 µg/ml streptomycin at 37 °C in a humidified atmosphere of 5% CO_2_. HeLa EFR3A-knockdown (KnD) and HeLa “scrambled” control were cultured in the same medium additionally supplemented with 2 µg/ml puromycin at 37 °C in a humidified atmosphere of 5% CO_2_.

### Detergent-resistant membrane

The detergent-resistant membrane (DRM) fraction was isolated from 25 × 10^6^ HeLa cells, which were washed with TNE buffer (10 mM Tris HCl, 150 mM NaCl, 5 mM EDTA, pH 7.5). A protease inhibitor cocktail was added and cells were resuspended in 300 µl of ice-cold DRM isolation buffer (10 mM Tris–HCl, 150 mM NaCl, 5 mM EDTA, 1% Triton X-100), incubated on ice for 20 min, occasionally vortexed and mixed with an equal volume of 80% sucrose in the same buffer. Finally, samples were gently applied on the top of a discontinuous sucrose gradient composed of 2.7 ml of 30% sucrose and 0.9 ml of 5% sucrose and ultracentrifuged for 16 h, at 165,000*g*, 4 °C, using an Optima L90K ultracentrifuge with a SW-60 Ti rotor (Beckman Coulter, Brea, CA, USA). After ultracentrifugation, ten fractions (420 μl) were collected from the top of the gradient.

### shRNA lentiviral particle’s transduction and transient transfections

HeLa EFR3A knockdown (KnD) and HeLa “scramble” were performed using EFR3A shRNA lentiviral particles (sc-77469-V) prepared by Santa Cruz Biotechnology (Dallas, TX, USA) according to the manufacturer’s protocol. HeLa cells were infected with shRNA lentiviral particles in a complete medium containing Polybrene^®^ (sc-134220) at a final concentration of 5 µg/ml. To select stable cell clones expressing shRNA, puromycin dihydrochloride concentration 2 μg/ml was used. Picked colonies were expanded and assayed for stable downregulation of the *EFR3*A gene. Transient transfections of cells were performed using CLB (Lonza, Basel, Switzerland) electroporation equipment according to the manufacturer’s protocol for HeLa cells, using program 4 as recommended. Cells were analyzed 48 h after transfection. Cells showed expression of EFR3A rescue at a constant level up to 72 h.

### Expression of recombinant proteins

His-tagged recombinant flotillin-2 was purified under denaturing conditions as described previously [26]. A human EFR3A cDNA clone in a bacterial expression vector pPB-N-His vector, containing a single N-terminal 6X-histidine tag, was ordered from genomics-online.com (Aachen, Germany). EFR3A was expressed in the *E. coli* strain NiCo21 (DE3) and purified under native conditions according to the previously published protocol with slight modifications. Initially, cells were precultured overnight at 37 °C, shaking at 180 rpm in 6 ml of LB medium containing kanamycin (35 µg/ml). Then, 400 ml of fresh LB medium with kanamycin (35 μg/ml) was inoculated with overnight preculture and grown at 37 °C to reach an optical density of the culture of 0.6 at 600 nm. Recombinant protein induction was carried out using IPTG at a final concentration of 0.5 mM at 18 °C with constant shaking (180 rpm) for 16 h. After this time, bacteria were collected by centrifugation at 10,000*g*/15 min and were resuspended in a lysis buffer, 10 mM HEPES, 500 mM NaCl, 1 mM PMSF, complete protease inhibitor cocktail, 10 mM imidazole, 0.5% Triton X-100, 25 U/ml nuclease OMNI, and 1 mg/ml lysozyme. The obtained suspension was homogenized via pressing through a needle (0.9 × 40 mm) followed by sonication in a Hielscher sonicator (UP100H) ten times for 0.5 s with 0.5 s intervals on ice, 80% amplitude. The bacterial lysate was centrifugated at 30,000*g* for 30 min. The obtained supernatant was mixed with Pierce high-capacity Ni-IMAC resin, EDTA-compatible, which was washed three times with a lysis buffer. The mixture was incubated at 4 °C for 3 h then placed into the column and washed three times with a wash buffer 1:10 mM HEPES, 500 mM NaCl, 10 mM imidazole, pH 8.0, followed by wash buffer 2:10 mM HEPES, 300 mM NaCl, 10 mM imidazole, pH 8.0, until the A_280_ dropped below 0.05. The protein was eluted with 300 mM imidazole 10 mM HEPES, 300 mM NaCl, pH 7.2. Peak fractions of the protein were analyzed by SDS–PAGE (10% gel). The gel was stained with Coomassie, or western blot was performed.

### Pull-down

Recombinant flotillin-2 was dialyzed against 0.1 M NaHCO_3_, 0.5 M NaCl pH 8.5 overnight at 4 °C. Flotillin-2 was mixed at 1:1 with the above buffer containing 2% octyl β-d-glucopyranoside, pH 8.5, and then covalently conjugated to CNBr Sepharose 4B according to the manufacturer’s protocol. Control resin was prepared by incubation with 0.1 M NaHCO_3_, 0.5 M NaCl, 1% octyl β-d-glucopyranoside, pH 8.5. To quench possibly remaining active groups, flotillin-2-conjugated and control Sepharose 4B resin were incubated with 0.2 M glycine, pH 8.0 for 16 h. Prepared resins were stored at 4 °C.

Resins prepared as above (70 µl) were centrifuged and then washed three times with a buffer (20 mM Tris pH 7.4, 150 mM NaCl, 1% octyl β-d-glucopyranoside, and 1 mM EDTA and protease inhibitor cocktail), by centrifugation at 1000*g* for 1 min at 4 °C. Both resins were incubated in 0.1% BSA in 20 mM Tris pH 7.4, 150 mM NaCl, 1% octyl β-d-glucopyranoside, 1 mM EDTA, protease inhibitor cocktail overnight at 4 °C, then washed ten times by centrifugation at 1000*g*/min with “lysis buffer” (50 mM Tris pH 7.5, 300 mM NaCl, 2% octyl β-d-glucopyranoside, protease inhibitor cocktail, and 2 mM EDTA). DRM fractions were mixed at a 1:1 ratio with the lysis buffer, kept on ice for 30 min (with occasional gentle vortexing), and incubated for 16 h with flotillin-2 Sepharose or control resins, with gentle shaking on a CappRondo CRR-08X blood mixer roller. After this time resins were washed eight times with lysis buffer, then bound proteins were eluted with 2 × SDS sample buffer (4% SDS, 20% glycerol, 10 mM EDTA, 100 mM DTT, 0.125 M Tris pH 6.8, and 0.01% bromophenol blue) and separated by SDS–PAGE (10% gel). Gels were stained with Coomassie and next the gel fragment containing protein bands of molecular mass greater than 60 kDa was cut out and subjected to MS/MS identification performed by the MS laboratory at the Institute of Biochemistry and Biophysics, Polish Academy of Sciences, Warsaw, Poland.

### Co-immunoprecipitation

9 × 10^6^ HeLa cells were solubilized in 20 mM Tris, 150 mM NaCl, 1 mM EDTA, 1% Triton X-100, 5% glycerol, 2% octyl β-*D*-glucopyranoside pH 7.4, and protease inhibitor cocktail for 30 min on ice and incubated with 5 μg of goat anti-flotillin-2 antibodies coupled to protein G Dynabeads at 4 °C overnight, with gentle shaking on a Rotator SB2 (Stuart). Nonimmune goat IgG (5 μg) was used as a control. The beads were washed with  PBS-T (Gibco, 0.1% Tween 20) and eluted with 5 × SDS sample buffer (10% SDS, 50% glycerol, 25 mM EDTA, 250 mM DTT, 0.3 M Tris pH 6.8, and 0.025% bromophenol blue) and separated by SDS-PAGE (10% gel) and analyzed by western blot with appropriate antibodies.

### MβCD treatment of cells

HeLa cells were grown as mentioned above for 48 h to confluency, then washed with PBS and incubated for 1 h at 37 °C with DMEM medium supplemented with 10% FBS, 2mM glutamine, and PBS in the presence (treated cells) or absence (control cells) of 8 mM MβCD. Then cells were treated with trypsin–EDTA 0.05% for 3 min at 37 °C. Finally, cells were collected by centrifugation at 200*g* for 5 min for further experiments.

### Cell cycle analysis

Cells were plated on 60 mm plates in triplicate and were cultured for 24, 48, or 72 h. The cells were treated with EDTA 0.05% for 3 min at 37 °C and collected in 5 ml round-bottom polystyrene test tubes. Then cells were washed with PBS by centrifugation at 200*g* for 5 min. The cell pellet was fixed by adding a few drops of cold 70% ethanol and stored at −20 °C. On the day of measurement, 2 ml of PBS was added to each cell pellet, then the suspension was centrifuged at 1000*g* for 5 min. The cell pellet was washed with 2 ml PBS again. Next, the pellet was suspended in 250 µl of 10 µg/ml RNase A in PBS and incubated for 45 min at room temperature. Then 8 µl of propidium iodide (1 mg/ml) was added to the cell suspension (250 µl) and incubated for 15–30 min (protected from the light). Before, the measurement samples were kept on ice. Measurement and analyses were performed on a NovoCyte flow cytometer (Agilent Technologies, Santa Clara, CA, USA). To quantify cell cycle distribution plots of FSC/SSC, PI-A/width, and PI-A/events were prepared using NovoExpress 1.2.4 software.

### FLIM analysis of living cells

FLIM was used to measure the fluorescence lifetime of the membrane-order sensitive probe, di-4 ANEPPDHQ (di-4) according to Owen et al. [29]. HeLa cells were grown in LabTek chambers in DMEM medium with 10% FBS and 2 mM glutamine. After 24 h cells were washed twice with HBSS (Gibco, Thermo Fisher Scientific, Amarillo, TX, USA) in 10 mM HEPES, pH 7.4, and stained with 2 μM di-4 in HBSS 10 mM HEPES, pH 7.4, for 5 min (the stock was 2 mM di-4 in DMSO). Cells were washed twice, and the measurements were performed in HBSS buffered with 10 mM HEPES, pH 7.4. Di-4 fluorescence lifetime microscopic images were obtained by time-correlated single-photon counting (TCSPC) using an LSM 510 META microscope (Carl Zeiss, Jena, Germany) equipped with a PicoQuant FLIM/FCS module (Berlin, Germany). Samples were excited at 470 nm and imaged with a 40 × WI objective (NA 1.2) using an LP 510 filter set. Acquisition time was adjusted to collect at least 1000 photons per pixel. Each pixel in the image was pseudocolored according to the average fluorescence lifetime. The images were analyzed in the PicoQuant Analysis program.

### GPMV isolation and FLIM analysis

Giant plasma membrane vesicles (GPMVs) were isolated from “scrambled” and KnD EFR3A HeLa cells, using vesiculation buffer [10 mM HEPES, 150 mM NaCl pH 7.25 and freshly added 2 mM CaCl_2_, 2 mM NEM (N-ethylmaleimide)] for 2 h at 37 °C according to the protocol [30]. GPMVs were stained with di-4 (2 μM) (10 min/RT) and placed in a covered 0.01% poly-l-lysine, sealed chamber prior to FLIM analysis as described above.

### svFCS measurements

Subconfluent cultures of HeLa cells were trypsinized and plated in number 35 × 10^3^ onto Lab-Tek 24 h before svFCS measurements. On the day of the experiment, cells were washed three times with HBSS buffered with 10 mM HEPES, pH 7.4 followed by 10 min incubation at room temperature in the dark, in 0.075 µM BSA, 0.075 µM Bodipy SM (Thermo Fisher Scientific, Waltham, MA, USA) in HBSS buffered with 10 mM HEPES. Next, cells were washed four times with HBSS in 10 mM HEPES, pH 7.4. Measurements were carried out using a custom-made svFCS system based on the Axiovert 200 M fluorescence microscope and 40 × WI objective (NA 1.2)" (Carl Zeiss, Oberkochen, Germany), according to the procedure described previously [16, 31]. Briefly, the 488 nm laser beam power was adjusted to 330 µW and the waist size was calibrated using a 2 nM rhodamine 6G solution. For the living cell analysis, all the measurements were performed under physiological conditions at 37 °C. The signal was recorded at an intensity of 2–4 μW and the data were collected in a series of 20 runs lasting 5 s each. The measurements were carried out on at least ten individual cells per spot size. Next, the generated autocorrelation functions (ACFs) were examined and analyzed by the IGOR Pro program. The data were fitted to a 2D lateral diffusion model and the average time diffusion time (*τ*_d_) was calculated. A single diffusion law was constructed from the measurements obtained at four different waist sizes.

### Rescue mutant

The shRNA-resistant control (rescue mutant EFR3A) was obtained through the generation of silent mutations within the coding sequence of the EFR3A protein, resulting in a synonymous amino acid product, but the protein mRNA was no longer a target for the shRNA. Such a prepared gene sequence was synthesized via Integrated DNA Technologies ITD (Redwood City, CA, 94065, USA). The vector obtained from the IDT sequence in pUCIDT (Amp) was subcloned into the p3XFLAG-CMV10 (Sigma) vector using NotI and KpnI restriction enzymes using the Quick Ligation kit (New England BioLabs). For svFCS control, EFR3A KnD cells (1.5 × 10^6^) were transfected with 1 µg of “empty” p3XFLAG-CMV10 vectors for 48 h at 37 °C in a humidified atmosphere of 5% CO_2_. For EFR3A “rescue” expression, EFR3A KnD cells (1.5 × 10^6^) were transfected with 1 µg of EFR3A “rescue” plasmidp3XFLAG-CMV10 EFR3A for 48 h at 37 °C in a humidified atmosphere of 5% CO_2_.

### Proliferation assay

Cell proliferation was evaluated by WST-1 cell proliferation assay (Takara) following the manufacturer’s instructions. Shortly HeLa “scrambled” and EFR3A KnD cells were seeded in 96-well plates at a density of 5000 cells per well and allowed to grow for 24 h. After adding the WST-1 cell proliferation assay reagent, cells were then incubated for 1 h at 37 °C and 5% CO_2_. Subsequently, absorbance was determined in a GloMax Discover microplate reader (Promega, Southampton, United Kingdom) at 450 nm.

### Wound healing assay

HeLa “scrambled” and EFR3A KnD cells were plated on 12-well plates and grown to 75–80% confluence in a complete medium and then they were serum-starved for 24 h. The cell-free gaps were created by an Ibidi Culture-Insert 3 well (ibidi GmbH, Gräfelfing, Germany). The cells were then treated with 50 ng/ml of EGF in a serum-free medium. The images (2464 × 2056 pixels) were captured at 24 and 48 h at 20× magnification using a Zeiss digital camera integrated with an Axio Vert.A1 inverted microscope (Carl Zeiss). The area of the wound was quantified using ImageJ software. The cell migration was expressed as the ratio of wound closure (R): R = [(A_0 h_ − A_∆ h_)/A_0 h_], where A _0 h_ is the area of the cell-free gap measured immediately after the insert was removed and A _∆ h_ is the area of the artificial wound measured after 24 or 48 h.

### Cytosolic Ca^2+^ concentrations

Subconfluent HeLa KnD EFR3A and HeLa “scrambled” cells were trypsinized and plated into Lab-Tek chambers 48 h before the experiment. A day before the experiment, the media were replaced with serum-free media, and cultures were incubated overnight (16–20 h). On the day of the experiment, cells were washed with HBSS buffered with 10 mM HEPES pH 7.4 and then the Fluo-4 Direct calcium assay kit was used according to the manufacturer’s protocol. Finally, cells were washed twice with HBSS as above. First, cells were analyzed without EGF. Next, EGF was added to the same wells to the final concentration of 50 ng/ml.

Fluorescence imaging was performed using a STELLARIS 8 system with a thermostated chamber at 37 °C and at excitation of 488 nm and emission within the range of 492–577 nm. Objective HC PL APO 86 ×/1.20 WI was used. Images were acquired every 1.3 s for 5 min. All image processing was performed using ImageJ Software (NIH). The fluorescence intensity of individual cells was obtained by defining a region of interest for each individual cell. The linear intensities were acquired from the “plot profile” over the entire 200 frames of the video.

### Western blotting and overlay assay

Protein samples in the amount of 15 μg were separated by SDS-PAGE and electrotransferred to 0.2 μm nitrocellulose membranes. Membranes were blocked with 5% nonfat milk in TBS-T, followed by overnight incubation at 4 °C with an appropriate primary antibody diluted as indicated in Table 1 in TBS-T. After washing three times with 0.1% Tween 20 in TBS (TBS-T), membranes were incubated with the appropriate horseradish peroxidase-conjugated secondary antibody, developed with chemiluminescence Radiance ECL kit, and visualized using an Azure 600 detector (Azure Biosystems, Dublin, CA, USA).

For overlay assay, purified recombinant EFR3A was subjected in the amount of 20 μg to SDS-PAGE and was transferred onto nitrocellulose membrane as was mentioned above and stained with Ponceau S. Next, membrane strips containing bands corresponding to molecular weight > 70 kDa were blocked with 5% dry milk in TBS-T overnight at 4 °C followed by incubation with increasing concentrations of recombinant flotillin-2 (purified flotillin-2 and dialyzed against TBS-T buffer), after which they were incubated with goat anti-flotillin-2 antibodies 1:1000. Finally, the blots were treated with HRP-conjugated anti-goat antibodies and a chemiluminescent reaction was developed and recorded as above.

### Cholesterol and protein determination

Analysis of the amount of cholesterol was performed using the Amplex Red cholesterol assay kit. The protein concentration was determined using the BCA protein assay kit. Subsequently, proteins in each collected fraction from the sucrose gradient were precipitated with 10% TCA prior to SDS-PAGE.

### Statistical analysis

The statistics were performed by one-way ANOVA and Student’s *t*-test using the Prism program (GraphPad, Boston, MA, USA). *p*-Value ≤ 0.05 was considered statistically significant.

## Results

### EFR3A is present in flotillin-2 interactome

**Fig. 1:**
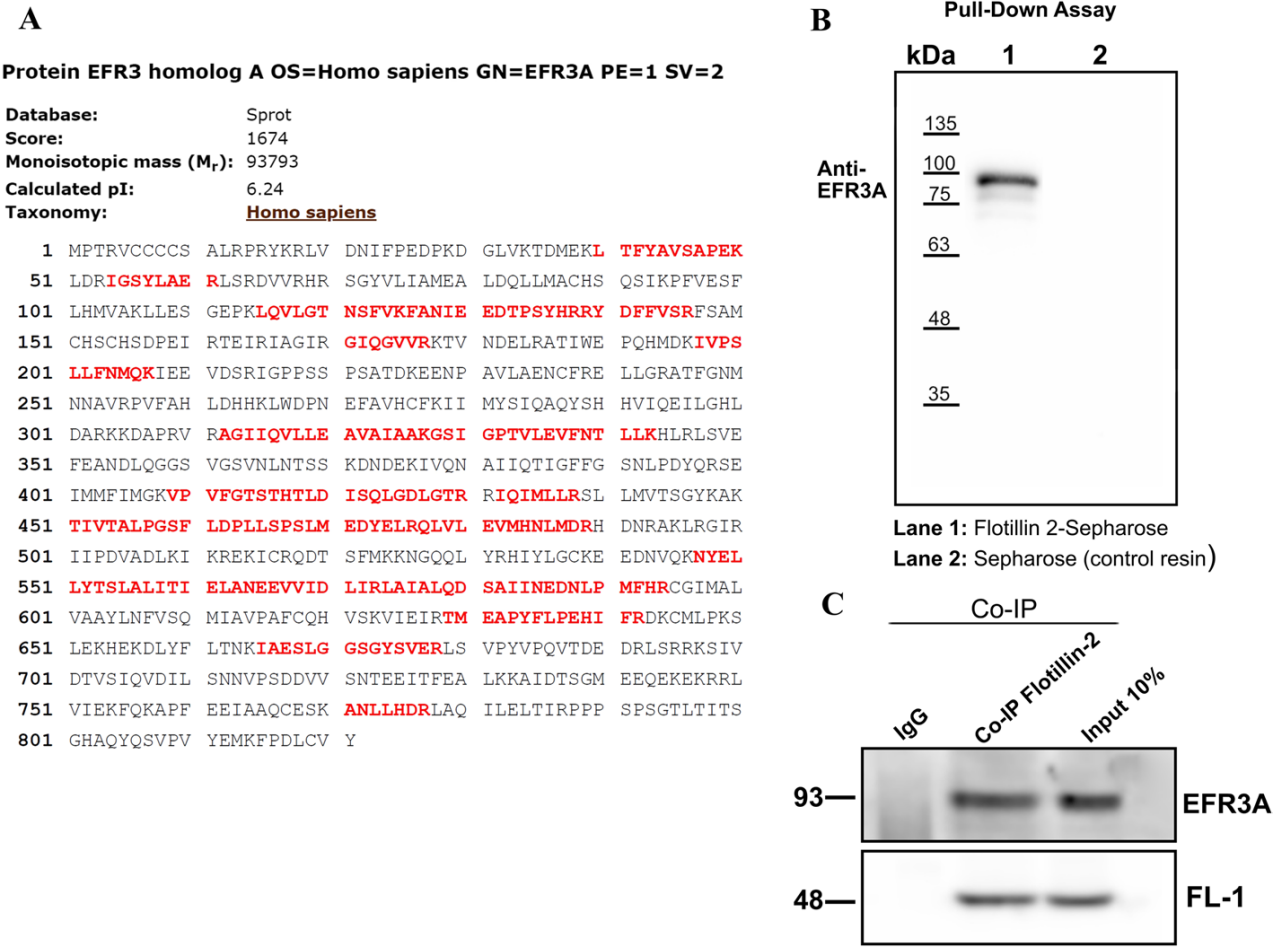
Flotillin-2 binds EFR3A in DRMs of HeLa cells. DRM fraction from HeLa cells diluted 1:1 with 2% octyl glucoside in 40 mM Tris–HCl, pH 7.4, 300 mM NaCl and protease inhibitor cocktail (Sigma–Aldrich) were incubated overnight with flotillin-2 Sepharose resin at 4 °C. Then the resin was thoroughly washed with the same buffer and suspended in sample buffer (20% SDS, 50% glycerol, 25 mM EDTA, 250 mM DTT, and 0.25 M Tris pH 6.8), boiled for 5 min, and subjected to SDS-PAGE. **A** Example of sequence coverage for EFR3A resulting from MS/MS identification of proteins bound to flotillin-2 Sepharose. SDS-PAGE gel fragment containing protein bands larger than 60 kDa was cut out and subjected to MS/MS identification (see also Additional file 1: Figure S1 and Table S1). **B** Western blotting of samples derived from a pull-down assay. Only bound fraction and unconjugated resin control are shown. Nitrocellulose was probed with an EFR3A antibody (Abnova). **C** Co-IP results, whole cell lysates of HeLa cells were incubated with 5 μg of anti-FL2 goat antibodies (Abcam) to protein G Dynabeads (Life Technologies) overnight at 4 °C with rotation, according to the manufacturer’s protocol. Nonimmune rabbit IgG (5 μg) was used as a negative control. Input means whole cell lysate proteins, 20 μg protein/well. Immunoprecipitated proteins were eluted in a sample buffer and analyzed by western blotting with rabbit anti-EFR3A antibodies and goat anti-flotillin-1 antibody as a positive control

As it transpired that MPP1 is not expressed at high levels in most human cell lines including HeLa cells [28], we anticipate that raft domain formation and regulation in these cells may depend on the interaction of flotillins with another protein partner(s). The first approach to resolve this issue involved pull-down experiments. For this purpose, we chose HeLa cells and recombinant bacterially expressed flotillin-2. As immobilization via His-tag to the metal-chelating resin was unsuccessful due to the high level of nonspecific binding, we tried CNBr-activated Sepharose 4B blocked with BSA. As a source of recognizable partners, we used the DRM fraction of HeLa cells. Several bound proteins were revealed via SDS-PAGE, and when the fragment of the gel containing proteins larger than flotillin (as shown in Additional file 1: Fig. S1) was subjected to MS/MS analysis, EFR3A protein was detected as a possible interactor of flotillin-2 (Fig. 1A and Additional file 1: Fig. S1B, Table S1). This protein was not present when the resin without bound flotillin-2 was used (see Additional file 1: Fig. S1C). It should be noted that this experiment includes MS/MS analysis of proteins larger than flotillins to avoid endogenous flotillins forming oligomers. These results were confirmed in the experiments in which flotillin-2-bound fractions were separated via SDS-PAGE and electrotransferred onto nitrocellulose membrane and probed with anti-EFR3A antibodies (Fig. 1B). Also, Co-IP using anti-flotillin-2 antibody resulted in the coprecipitation of EFR3A (Fig. 1C), in contrast to the control sample not showing specific immunoreactivity against EFR3A protein.

**Fig. 2:**
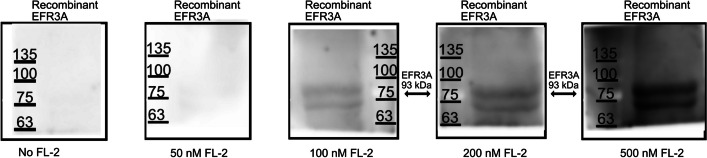
Bacterially expressed recombinant EFR3A binds to recombinant flotillin-2 in vitro, in the overlay assay. Recombinant EFR3A protein was purified on metal ion chelating resin (Ni^2+^) and subjected to SDS-PAGE followed by transfer onto nitrocellulose. Then, membrane strips containing bands corresponding to molecular weight larger than 70 kDa were incubated with increasing concentrations of recombinant flotillin-2, followed by incubation with goat anti-flotillin-2 antibodies (Abcam) and secondary donkey anti-goat antibodies (Santa Cruz)

In the next experiment we wanted to test whether this interaction could be demonstrated in vitro, on recombinant, bacterially expressed EFR3A and flotillin-2. EFR3A was purified using Ni^2+^ resin and blotted onto nitrocellulose and the latter was incubated with increasing concentration of flotillin-2 solution in the presence of nonionic detergent. Then, membrane strips were incubated with anti-flotillin-2 antibodies followed by secondary horseradish peroxidase-conjugated antibodies and visualized. As purified recombinant EFR3A undergone substantial proteolysis during expression and purification, which we could not have prevented, we confirmed its identity via western blotting with anti-EFR3A, anti-HisTag antibodies, and also via MS/MS sequencing (Additional file 1: Fig. S4 A–C). Results of overlay assay using the entire lane may point to N-terminal fragment as a putative binding site for flotillin-2 in EFR3A (Additional file 1: Fig. S4D, E). Overall the results shown in Fig. 2 may indicate the interactions of these two proteins in vitro.

Thus, we infer that flotillin-2 interacts with the EFR3A protein, most likely within the plasma membrane (represented by DRMs) of studied cells. It should be stressed that this interaction has been reported neither in the literature nor in databases yet (see Additional file 1: Fig. S2).

### Is EFR3A raft domain associated?

The next question concerns the raft association of the newly discovered flotillin-2 interacting protein EFR3A. Flotillins are always seen associated with rafts, including DRM fractions. These proteins are present in the low-density fraction of the Triton X-100 extract of cells or membranes even if the cells have been treated with raft domain disrupting agents, e.g., by depleting membrane cholesterol, such as methyl-β-cyclodextrin, or by decreasing the level of MPP1, the protein which was previously shown to participate in raft domain organization [25].

**Fig. 3:**
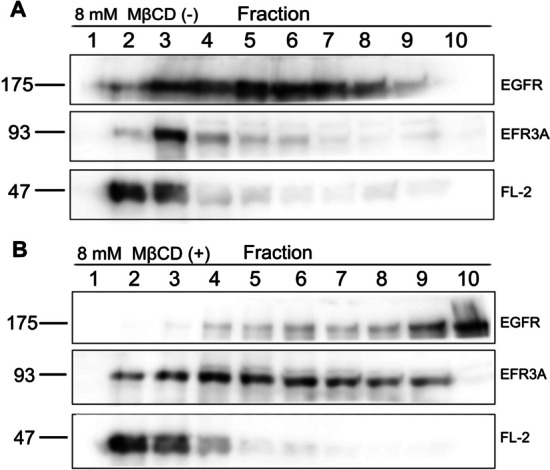
EFR3A is present in the DRM fraction and its localization in the DRM fraction is sensitive to the removal of cholesterol from the HeLa cell plasma membrane. **A** Western blotting of sucrose step gradient fractions obtained after ultracentrifugation of cold 1% Triton X-100 extract (30 min on ice) from HeLa cells. 25 × 10^6^ cells were used. Then, fractions were collected and proteins were precipitated with 10% TCA and analyzed with appropriate primary and secondary antibodies. **B** Western blotting of the Triton extract from cells treated with 8 mM β-methyl-cyclodextrin shows that localization of EFR3A in the DRM fraction is sensitive to the removal of cholesterol; for comparison, anti-EGFR, and anti-flotillin antibodies reactions are shown. For total protein and cholesterol profiles see Additional file 1: Fig. S3

Data shown in Fig. 3 indicate that EFR3A is detected only in DRM fractions from HeLa cells. Moreover, upon partial cholesterol extraction from living cell plasma membrane, which is known to disrupt the raft domain (see also Additional file 1: Fig. S3), EFR3A can be detected in the middle and high-density fractions of the sucrose gradient which may suggest raft domain association. This is also true for EGFR. As EGFR localization in the raft domain, particularly in the DRM fraction, is concerned, we have to note that localization of this receptor in the raft domain may differ in various studies, depending on the protocol of DRM isolation. For example, Puri et al. suggested the almost complete absence of EGFR from DRM fraction and raft domain. The main difference between the mentioned and our experimental protocol was the fact that the authors isolated DRMs from serum-starved HeLa cells and observed EGFR recruitment upon EGF treatment. The effect was readily detected at an EGF concentration of 1.5 ng/ml, which might explain the difference [32].

### Involvement of EFR3A in lateral membrane organization

**Fig. 4:**
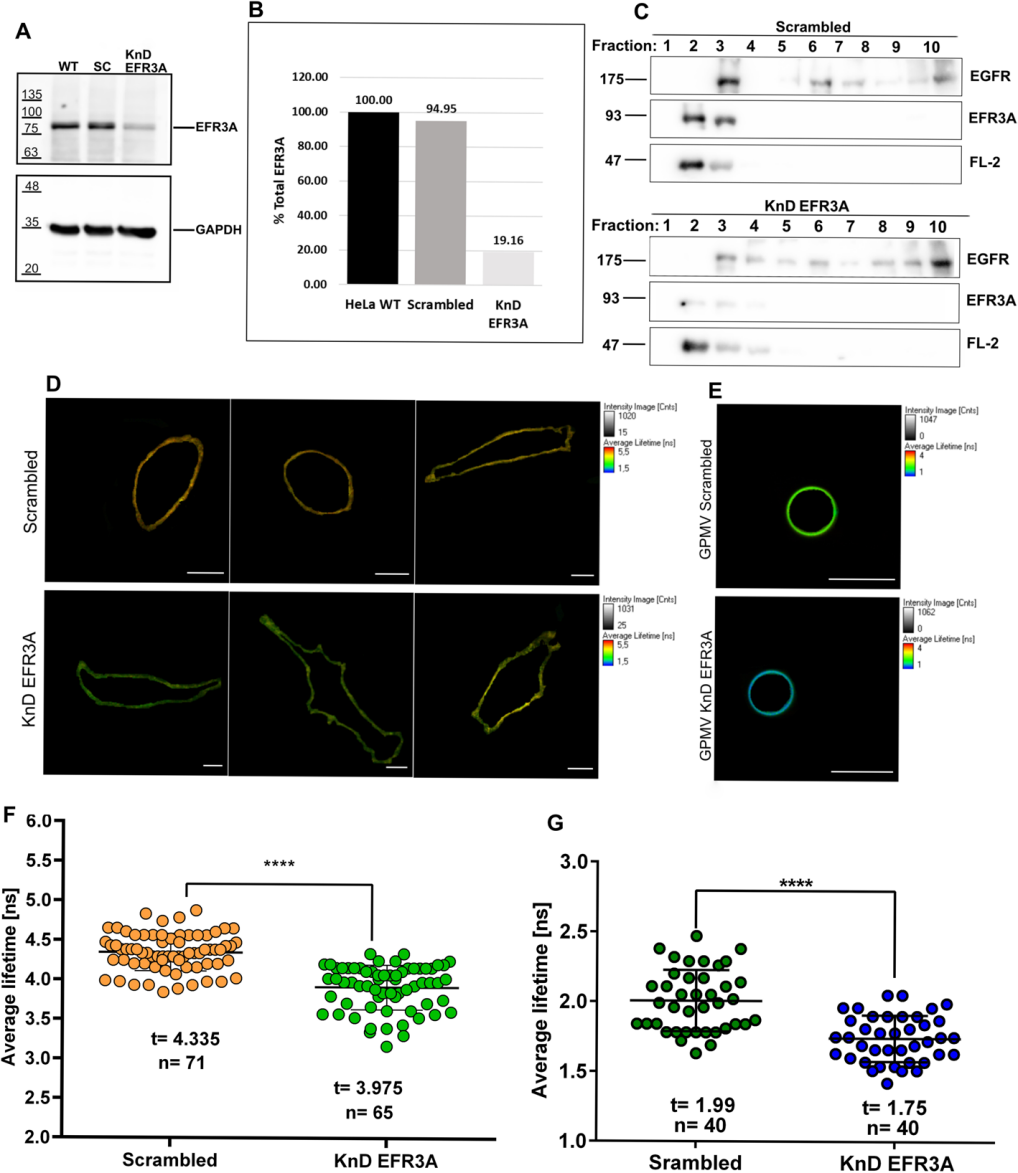
Silencing expression of the *EFR3A* gene in the Hela cell line induces changes in lateral membrane organization. Stable cell lines were obtained using EFR3A shRNA lentiviral particles or “scrambled” shRNA lentiviral particles as described in the Methods section. **A** Cell extracts of control or transduced cell lines were submitted to SDS-PAGE and western blotting probed with anti-EFR3A antibodies. GAPDH visualization was used as a loading control. **B** Quantitation of the EFR3A fractions shown in **A**. **C** Marked decrease of EFR3A level in cells is accompanied by a substantial reduction of EGFR in the DRM fraction. **D** Examples of FLIM images of the cell plasma membrane and **E** GPMVs derived from *EFR3A* KnD and “scrambled” cells. **F** and **G** Quantitation of FLIM data of *EFR3A* KnD and “scrambled” cells and corresponding GPMVs. For FLIM measurements of the fluorescence lifetime of the membrane-order sensitive probe, di-4 ANEPPDHQ (Invitrogen) control “scrambled” and *EFR3A* KnD HeLa cells were grown in LabTek chambers in DMEM medium with 10% FBS. After 24 h cells were washed twice and stained with a 2 μM di-4 probe in DMEM medium for 5 min. Cells were washed twice in HBSS buffer with 10 mM HEPES pH 7.4 and measurements were performed. Giant plasma membrane vesicles (GPMV) were generated from *EFR3A* KnD and “scrambled” cells as mentioned in the Methods section and stained with di-4 (2 μM) as above. The lifetime values of wild-type (WT) cells did not differ from “scrambled”, so we did not include them for clarity

To gain insight into the possible role of EFR3A in membrane raft domain organization and regulation we obtained *EFR3A* KnD or “scrambled” HeLa cells using EFR3A shRNA lentiviral particles or “scrambled” shRNA lentiviral particles, respectively. As can be seen in Fig. 4A, B, the level of EFR3A was markedly (by ~ 80%) decreased compared with control (untreated) or “scrambled” cells. Moreover, the decrease in EFR3A protein in KnD cells was accompanied by a marked decrease of EGFR in the DRM fraction (Fig. 4C).

FLIM analysis of the di-4 probe in *EFR3A* KnD cell plasma membranes (Fig. 4D, F) indicated that silencing of EFR3A expression resulted in a statistically significant decrease in membrane order, which is reflected by 0.3 ns of fluorescence lifetime and is in good agreement with previously obtained results for silencing of MPP1 expression in erythroid cells [25, 33]. Similarly, also significant differences in membrane order changes were recorded when FLIM observation was carried out on GPMVs derived from “scrambled” and KnD cells (Fig. 4E, G). It is plausible that these differences stem from changes in lipid bilayer properties and may point to an important role of EFR3A in raft domain organization.

**Fig. 5:**
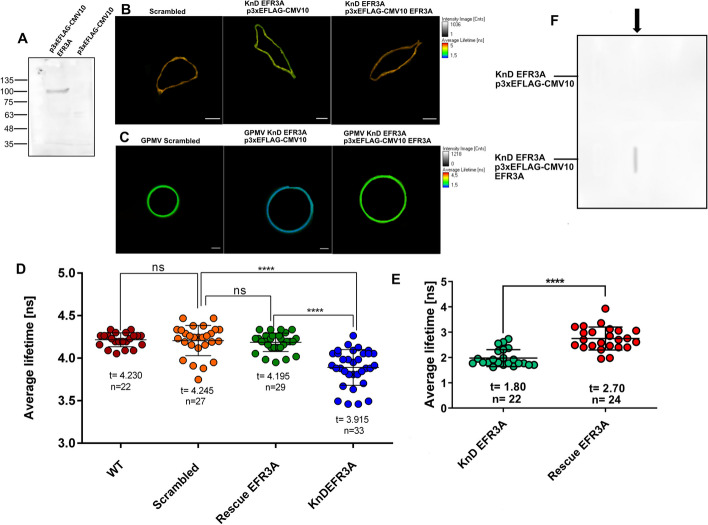
“Rescue expression” of *EFR3A* in KnD HeLa cells. **A** Western blotting of total protein extracts of *EFR3A* KnD HeLa cells transfected with p3xEFLAG-CMV10 *EFR3A* (lane 1) and control p3xEFLAG-CMV10 plasmids (lane 2). Anti-FLAG antibodies (Merck) were used as primary antibodies. **B** Representative di-4 FLIM images of control, “scrambled” cell (left), EFR3A KnD cell transfected with an empty vector (middle), and “rescue transfected” (right). **C** Examples of di4 FLIM images of GPMVs generated from control, “scrambled” cells (left), EFR3A KnD cells transfected with an “empty” vector (middle), and EFR3A KnD cells transfected with a “rescue” plasmid p3xEFLAG-CMV10 EFR3A. **D** and **E** Quantitative distribution of fluorescence lifetime values obtained from FLIM analyses of di-4 labeled cells. **D** Plasma membrane and GPMVs. **E** FLIM analyses presented here were performed 48 h following transfection. **F** Dot-blot analysis of GPMVs derived from “rescue”-transfected EFR3A KnD cells using an anti-FLAG antibody. GPMVs were induced using a buffer containing NEM and CaCl_2_ 48 h after transfection. Then, from the collected vesicles, proteins were precipitated with 10% TCA, washed, and resuspended in a reducing buffer containing DTT. Samples were applied on nitrocellulose using a dot blotter and probed with anti-FLAG antibodies

Using KnD cells raises the question of whether the observed effects are not the results of so-called off-target effects, i.e., do they really result from silencing of a particular gene? Apart from the “scrambled” control we employed transfection with a rescue plasmid, i.e., a vector carrying the *EFR3A* sequence with mutations making it resistant to silencing shRNAs also carrying a FLAG-tag sequence. As shown in Fig. 5A, transfection of *EFR3A* KnD HeLa cells with this vector resulted in the presence of FLAG-tagged protein of molecular mass close to 95 kDa, which was absent in control cells (transfected with the empty vector). When transfected with the rescue vector, cells, and cell-derived GPMVs were subjected to FLIM (Fig. 5B, C) analysis. It was found that the membrane order returned to “normal,” i.e., was similar to the “scrambled” control and significantly different from KnD cells (Fig. 5D). The presence of EFR3A protein in GPMVs obtained from cells transfected with the rescue vector was confirmed by dot blot analysis, as shown in Fig. 5F.

### EFR3A determines molecular diffusion and confinement of the lipid raft marker sphingomyelin

Marguet et al. [16] invented a spot variation fluorescence correlation spectroscopy (svFCS) method which can provide insight into molecular diffusion of molecules of the plasma membranes of living cells with high spatiotemporal resolution. Here, the diffusion time as a function of the varying fluorescence detection area (”waist”) is measured, which allows the calculation of the t_0_ parameter. The latter enables distinguishing membrane molecules undergoing free, unlimited diffusion (t_0_ = 0), temporally confined to membrane domains (t_0_ > 0) or experiencing diffusion limited by the membrane skeleton/cytoskeleton (t_0_ < 0) [16].

**Fig. 6:**
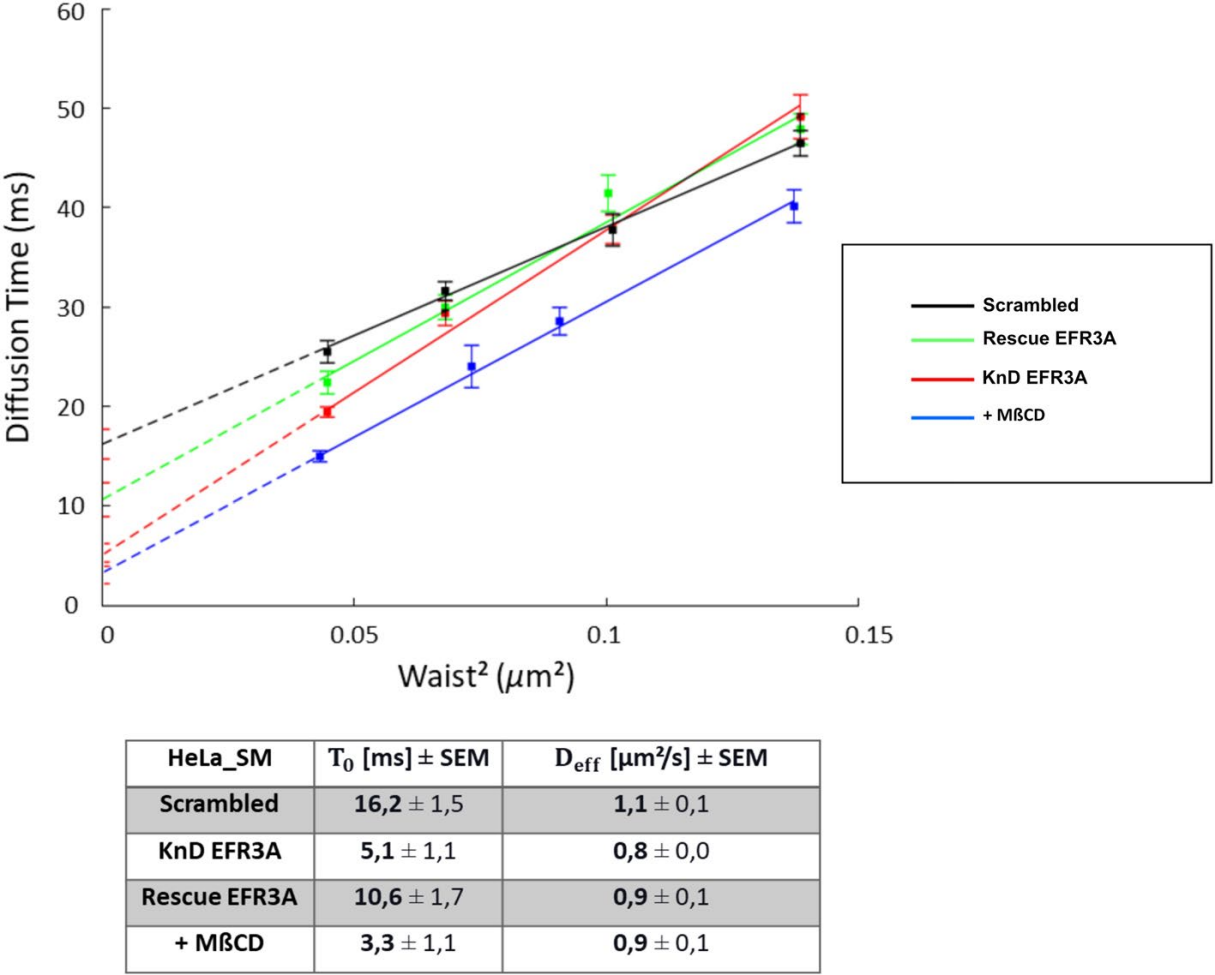
Mobility of the raft probe in *EFR3A* KnD partially returns towards normal in “rescue”-transfected *EFR3A* KnD HeLa cells svFCS measurements. **A** Diffusion time as a function of waist surface area. Black line: control HeLa “scrambled”, red line: *EFR3A* KnD, and green line: “rescue”-transfected *EFR3A* KnD HeLa cells. Cells were grown in LabTek chambers in DMEM medium with 10% FBS. After 24 h cells were washed three times and stained with 0.075 μM BODIPY-SM in HBSS with 10 mM HEPES and 0.075 μM BSA medium for 10 min. Cells were washed three times and measurements were performed in HBSS buffer with 10 mM HEPES, pH 7.4. For comparison, wild-type cells were treated with 8 mM β-methyl cyclodextrin for 1 h at 37 °C **B**. T_0_ and apparent diffusion coefficient. SEM, standard error of the mean

When svFCS experiments using a raft domain probe, i.e., BODIPY-SM, on *EFR3A* KnD HeLa cells were performed, a decrease in positive t_0_ value compared with the control “scrambled” cell line could be observed (5.1 versus 16.2 ms), indicating a marked decrease in raft domain confinement of the probe (Fig. 6A, B). Moreover, transfection of *EFR3A* KnD cells with a rescue vector caused t_0_ to return to higher values (10.6 ms). This may indicate an increase in the raft domain immobilization of the fluorescent probe. As a control experiment, “scrambled” cells were treated with MβCD to extract cholesterol from the plasma membrane and disrupt raft domains. Such treatment radically decreases raft domain confinement of the fluorescent probe (blue line). It should be noted that the raft probe we used (BODIPY-SM) was proven to represent raft domain constituents in various types of living cells by us (see e.g., [34–36] and by others [37]).

Overall, the results from the svFCS experiments confirm our hypothesis that EFR3A, being a flotillin-interacting protein, is involved in organizing the raft domain in the plasma membrane of HeLa cells. They are in agreement with the data from FLIM experiments and essentially resemble the data obtained previously by our team for MPP1 in erythroid cells. Therefore, it may be concluded that EFR3A in general fulfills the role of a membrane raft organizer protein.

### Cell signaling

It is generally accepted that membrane raft domains function as signaling and sorting platforms for a large number of surface receptors and other proteins involved in the regulation of various physiological processes. In particular, they play a prominent role in many signal transduction pathways, which regulate proliferation, susceptibility to apoptosis, survival, cell adhesion, and migration [6].

**Fig. 7:**
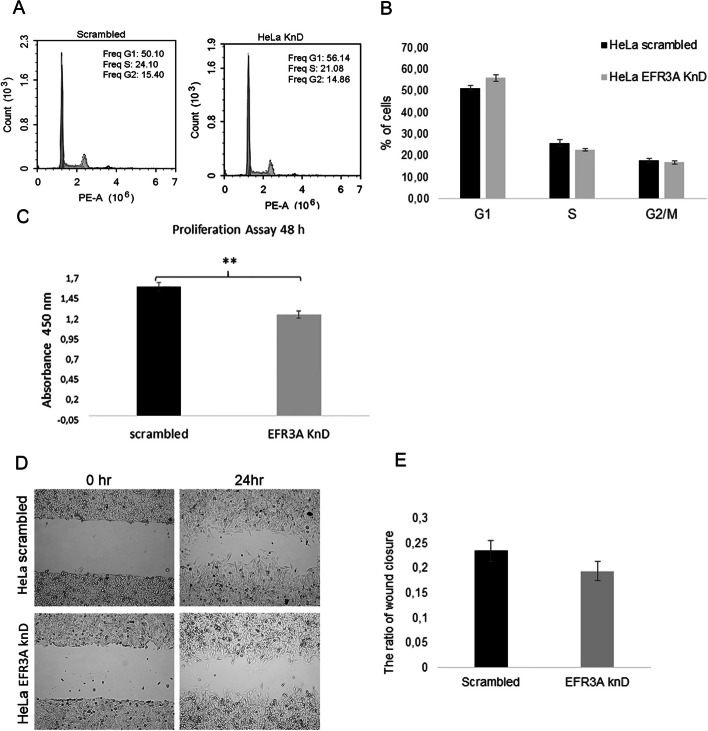
Silencing of *EFR3A* gene expression affects G1 and G2/M length and proliferation but not cell mobility. **A** Cell cycle phase distribution in HeLa cells was evaluated via PI staining followed by flow cytometry presented as histograms. **B** Quantitation of flow cytometry analysis. Error bars represent the SEM of three independent experiments. *p < 0.05. The percentage of G1 phase cells increased within 48 h to 56%. **C** Cell proliferation was evaluated by WST-1 staining according to the Methods section. **Indicates a *p* ≤ 0.01 compared with control cells. **D** Representative images from the wound healing assay showing changes in HeLa cells with decreased *EFR3A* gene expression in comparison with control “scrambled” cells under stimulation with EGFR (50 ng/ml) for 48 h. **E** Quantitative analysis from the wound healing assay in the form of a bar graph showing the ratio of wound closure in EFR3A KnD versus “scrambled” cells. The observed reduced motility in cells with decreased expression of EFR3A in comparison with “scrambled” cells was found to be statistically insignificant (*p*-value = 0.158)

As we observed changes in the plasma membrane order in HeLa cells with reduced expression of the EFR3A protein, we further measured the cell cycle phase distribution of HeLa *EFR3A* KnD cells by propidium iodide (PI) staining. As shown in Fig. 7A, B, a higher proportion of *EFR3A* knockdown cells were arrested in the G1 phase compared with “scrambled” control cells. The percentage of G1 phase cells with decreased expression of EFR3A increased significantly (*p* < 0.05) from 50 to 56% within 48 h, which suggests a potential role of EFR3A-dependent membrane organization in cell cycle progression. These data are supported by the results obtained in the proliferation assay, where EFR3A knockdown significantly reduces HeLa cell proliferation as shown in Fig. 7C.

Next, we tested the mobility of *EFR3A* KnD cells via a wound-healing assay. In these experiments, we observed that the motility of the cells with reduced *EFR3A* gene expression was slightly lower than that of the control “scrambled” cells (Fig. 7D, E). It should be noted, however, that changes in the cell motility were not large and were statistically nonsignificant.

It is well known that EGF receptors, whose function has been implicated in the regulation of cellular events including proliferation, survival, differentiation, and migration [6], are localized in membrane rafts and their activity depends on such localization. Phospholipase Cγ is the only one of the four PLC subfamilies activated downstream of receptor tyrosine kinases (via the SH2 domain present in the PLC molecule) after being recruited to membrane rafts. Thus, it was of interest whether silencing of *EFR3A* expression in HeLa cells would affect activation (phosphorylation) of EGFR and PLCγ1. It should be noted that localization of EGFR in the DRM fraction was strongly affected by silencing *EFR3A* gene expression (see Fig. 4C), which, bearing in mind all reservations concerning the relationship of the DRM fraction and membrane rafts, might indicate a diminution of raft domains.

**Fig. 8:**
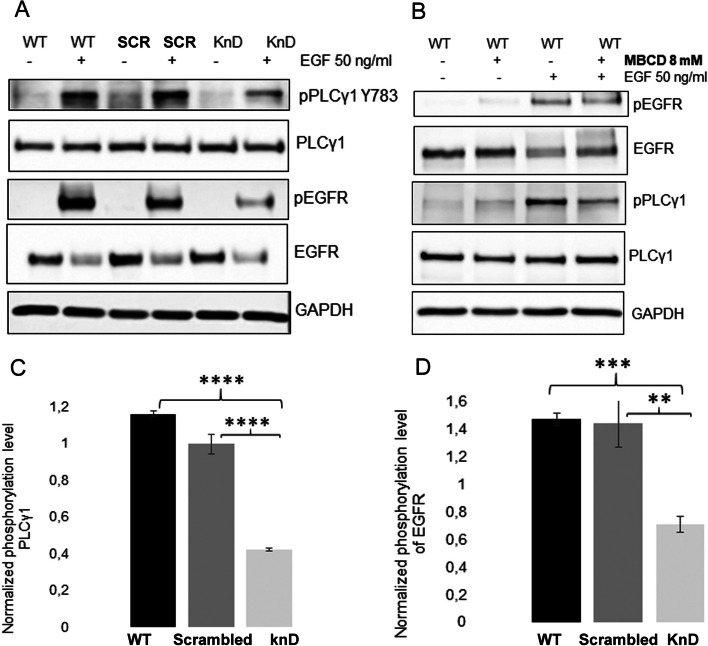
Silencing of *EFR3* gene expression affects PLCγ1 and EGFR phosphorylation. **A** Effect of *EFR3A* gene silencing on activation of EGF-induced EGF receptor and phospholipase PLCγ1 in HeLa cells via western blotting analysis. **B** Effect of MβCD on EGFR and PLCγ1 phosphorylation upon EGF treatment of WT HeLa cells. Fifteen micrograms of cell lysates was loaded on the 10% SDS-PAGE gel. **C** and **D** Quantitative analysis of data presented in **A** as bar graphs of fold change calculated as the ratio of relative levels of phospho-PLCγ and pEGFR normalized to the corresponding PLCγ1 and EGFR signal respectively. Significance values were calculated by paired Student’s *t*-test for **p < 0.01, ***p < 0.001 and ****p < 0.0001

Western blot analyses of the phosphorylation levels of EGFR and PLCγ1 upon stimulation with 50 ng EGF were performed. Indeed, when activated with EGF, the phosphorylation of EGFR was markedly diminished in *EFR3A* KnD as compared with “scrambled” and wild-type cells (Fig. 8A, D). The observed decrease in total EGFR could be a result of increased internalization of the receptor via clathrin-coated pits and targeting for degradation [38]. A similar effect, i.e., marked reduction in phosphorylation of PLCγ1 upon EGF stimulation in *EFR3A* KnD cells as compared with control cells (Fig. 8A, C) was observed. In the control experiment, when wild-type HeLa cells were treated with MβCD to extract cholesterol from the plasma membrane and disrupt raft domains, a decreased phosphorylation level in both EGFR and PLCγ1 was also observed (Fig. 8B). These experiments suggest that “disruption” of the membrane raft domain via reducing the amount of EFR3A in the cells leads to the decrease in EGFR and PLCγ1 phosphorylation.

### EGF-induced Ca^2+^ level

It has been known for a long time that membrane rafts organize the EGF receptor and that its activation induces intracellular Ca^2+^ increase which occurs via the activation of numerous channels, among them K–Ca^2+^, Ca^2+^, and Cl–Ca^2+^, which are involved in the signaling pathways regulating cell proliferation and migration or apoptosis (for a review see e.g., [39]).

**Fig. 9:**
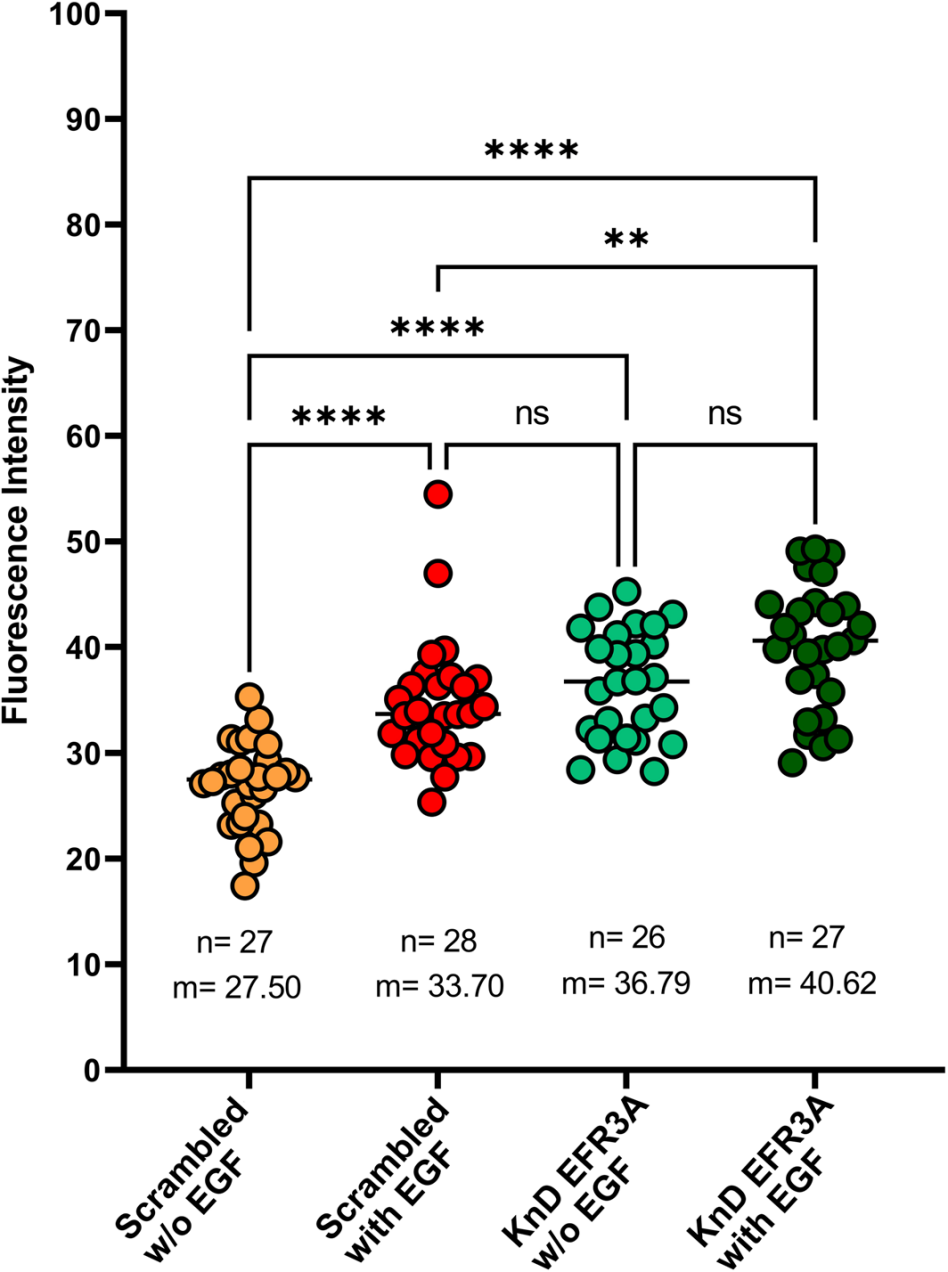
Silencing of *EFR3* gene expression affects EGF-induced Ca^2+^ influx. Fluo-4 Direct calcium assay kit was used according to the manufacturer’s protocol. First, cells were analyzed without EGF. Next, EGF was added to the same wells to the final concentration of 50 ng/ml. Fluorescence was acquired using a STELLARIS 8 system with a thermostated chamber at 37 °C and at excitation of 488 nm and emission within the range of 492–577 nm. Objective HC PL APO 86x/1.20 water numerical aperture 1.2 was used. Images were acquired every 1.3 s for 5 min. All image processing was performed using ImageJ Software (NIH). The fluorescence intensity of individual cells was obtained by defining a region of interest for each individual cell. The linear intensities were acquired from the “plot profile” over the entire 200 frames of the video. For further details see Methods and Additional file 2: Movie 1, Additional file 3: Movie 2

As shown in Fig. 9 (see also Additional file 2: Movie 1, Additional file 3: Movie 2), the level of this ion in stimulated control “scrambled” cells increases upon stimulation with EGF, which was expected. However, as in *EFR3A* KnD cells, the basic, unstimulated level is high, reaching almost that of the stimulated control cells; the result of stimulation is not evident, suggesting dysfunction of Ca^2+^ control systems.

## Discussion

We are interested in the molecular mechanism(s) governing membrane raft domain organization and regulation. In light of recent literature, it seems almost certain that membrane proteins participate in these processes [2, 15], although unambiguous data are rather scarce. As mentioned above, flotillins occur in the DRM fractions derived from every cell line we have tested. It was true even in raft domain-disorganizing conditions such as MβCD treatment, inhibition of protein palmitoylation, or MPP1 knockdown in erythroid cells [25]. Therefore, we assume that flotillin (probably flotillin-1/-2 heterodimer or a higher oligomer) is a major component of preexisting raft precursors composed of a few proteins and lipids, which upon interaction with a protein raft domain organizer such as MPP1 in the case of erythroid cells become resting state rafts, i.e., larger (~ 20 nm in diameter), more stable (*τ*_1/2_ ~ 1 s) and functional [7]. The lipid bilayer included within these entities becomes less fluid (liquid-ordered-like). Taking into account the above, MPP1 was previously considered to fulfill the role of a membrane raft domain organizer. The following criteria of membrane raft domain organizer protein could be established. Namely, silencing of candidate protein-encoding gene expression: (1) reduces membrane order and increases the mobility of lipid molecules as measured by several methods and (2) affects cellular signaling pathways. Moreover, the candidate protein (3) interacts with moderate-to-high affinity with flotillins in cells and in solution [25–27, 33, 34]].

The lack of high-level expression of *MPP1* in most human-derived cell lines suggests that other proteins might also fulfill this role. Taking the above-mentioned criteria into consideration, our assumption was that the candidate protein would bind flotillins. Thus, our pull-down approach to fish out the candidate protein on immobilized flotillin seemed the obvious choice. Flotillin-2 was chosen as it was previously confirmed that basic structural units of flotillins within microdomains are homo- and heterotetramers dependent on the presence of flotillin-2 [40]. Flotillin-2 is also considered to be more tightly associated with the membranes than flotillin-1 due to additional myristoylation. Our previous reports [26, 34] show that flotillin-2 indeed forms clusters at the plasma membrane and is a primary partner of cluster arranging protein, MPP1. Moreover, the mobility of these clusters strongly depends on MPP1-flotillin interactions. Therefore, we focus primarily on flotillin-2 as a potential partner for EFR3A. Our attempts were performed on immobilized flotillin-2 against the DRM fraction of HeLa cells. Among the bound proteins shown via MS/MS technique, EFR3A was repeatedly detected. Moreover, western blotting using anti-EFR3A antibodies to detect fished-out protein and Co-IP experiments confirmed results obtained via MS/MS technique. It should be mentioned that although EFR3A’s presence in the DRM fraction might suggest its connection to the rafts, it cannot be used as a criterion as many controversies were raised regarding the relationship between DRMs and raft domains [41]. However, it was proved as a very useful starting point in our search for a raft domain-organizing protein [2]. EFR3A was also found in the DRMs from other human cell lines such as LNCaP and PC-3 and was pulled down on immobilized flotillin-2 (unpublished data). Also, as shown in Additional file 1: Figs. S5 and S6 KnD of the *EFR3A* gene also affected the membrane order of other cell type, such as MCF7, although the difference in the probe lifetime was smaller but still statistically significant. Moreover, the presence of EFR3A in the DRM fraction appeared sensitive to the removal of cholesterol, which might additionally support its relationship with rafts. The interaction of flotillin-2 with EFR3A was also confirmed in vitro in an overlay experiment by using bacterially expressed recombinant proteins. Therefore, criterion 3, i.e., interaction of the candidate protein with flotillins, seems to be fulfilled. It should be noted that this interaction has not been reported yet in the literature and in the interactome databases (see e.g., Additional file 1: Fig. S2).

The effect of silencing the candidate gene expression (criterion 1), which we tested by using lentiviral technology on HeLa cells, seems to provide convincing evidence supporting our notion that EFR3A might serve as a raft domain organizing factor. As a result of silencing the *EFR3A* gene, a decrease in membrane order was observed, as also observed in the case of GPMVs derived from KnD cells, although the absolute amplitudes were different. This was similar to the results on MPP1, which we observed previously on HEL cells [25, 34]. The results of rescue expression indicate that the above-reported consequences were not the result of an off-target effect but were specific results of *EFR3A* KnD.

Moreover, svFCS data indicate changes in diffusivity of the raft probe BODIPY-SM in *EFR3A* KnD cells while comparing with control cells (which in all experiments were HeLa cells transduced with a lentiviral vector bearing the “scrambled” sequence). The value of t_0_ changes significantly towards unrestricted diffusion of BODIPY-SM due to the partial deprivation of EFR3A. Moreover, t_0_ was shifted back towards the higher values in cells transfected with the rescue vector expressing wild-type *EFR3A*, again supporting our notion that the observed effect of *EFR3A* knock-down is specific and not a result of an off-target effect. Similar results were obtained recently for *MPP1* KnD HEL cells [34]. Furthermore, those results confirmed similar changes in diffusivity also for GPI-anchored membrane glycoprotein, which is known as a raft protein. The mechanism of these changes is still unknown, but we can speculate that the clustering of flotillins may result in the recruitment of other typical raft lipids and proteins.

In summary, silencing of *EFR3A* gene expression may support our hypothesis that EFR3A is a constitutive component of the raft domain and may be involved in the organization and regulation of this domain.

It is known from many studies that raft domains are involved in cellular signaling. For example, silencing of *MPP1* gene expression was responsible for changes in insulin-dependent ERK1/2 signal transduction and H-RAS regulation [25, 42, 43]. Data presented here indicate a rather small effect of *EFR3A* knockdown on the cell cycle and mobility and a small but significant effect on the proliferation of HeLa cells. On the other hand, significant effects on EGFR phosphorylation, EGF-induced PLCγ phosphorylation, and Ca^2+^ influx were observed. It is well known that EGFR is localized within rafts and its function is raft-dependent [44–46] (for a review see [11]). Also, PLCγ1 activation may take place with the participation of major kinases belonging to the RTK superfamily [47] such as PEGFR, VEGFR, EGFR, FGFR, and Trk.

Observed down-regulation of EGF-induced PLCγ phosphorylation is apparently related to the disruption of signaling via EGFR. The functional connection of EGFR and PLCγ and raft domains has been known for some time [48], so the inhibition of PLCγ phosphorylation as a result of at least partial disruption of raft domain and EGFR release/lack of phosphorylation could confirm our hypothesis considering EFR3A as a raft-organizing protein. Knockdown of *EFR3A* resulted in the distortion of Ca^2+^ equilibrium, so the effect of EGFR stimulation was not pronounced. This result also confirms that the change in the basic level of intracellular Ca^2+^ could be a result of partial raft domain disruption. However, this could be a result of various influences on raft domain-dependent calcium ion transporters, among them plasma membrane Ca^2+^ATP-ases [49] or a variety of Ca^2+^ channels [50, 51].

Altogether, our results suggest hitherto unreported flotillin-2–EFR3A interaction, which by several criteria may appear responsible for membrane raft organization and regulation. This shows the participation of this interaction in the regulation of multiple EGFR-dependent cellular processes.

**Fig. 10:**
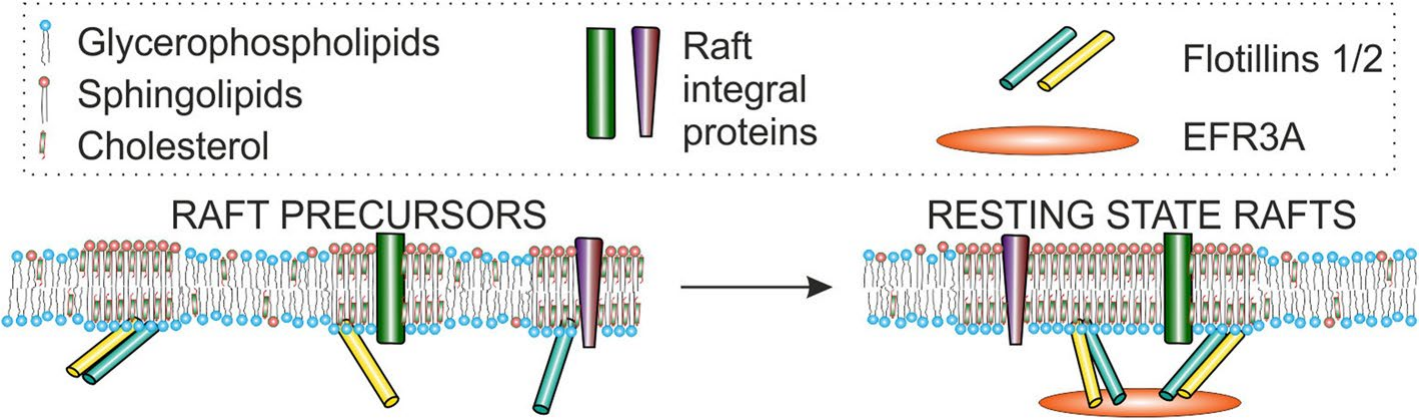
Proposed model of EFR3A participation of raft domain regulation. Flotillin (most likely flotillin-1/-2 heterodimer) is a member of preexisting unstable protein–lipid complexes (τ1/2< 0.1 ms; < 10 nm in diameter) composed of few proteins and lipids which upon interaction with a protein–raft domain organizer, such as EFR3A, cluster into raft domains [resting state rafts, i.e., domains ~ 20 nm in diameter, which are considered more stable (*τ*_1/2_ ~ 1 s) and functional]. Protein clustering modulates membrane bilayer physical properties, such as fluidity and local diffusion

Our hypothesis of EFR3A function as a raft domain-organizing factor is analogous to that proposed for MPP1. We anticipate that the flotillin binding described here is responsible for oligomerization and/or stabilization of preexisting unstable protein–lipid complexes (τ1/2< 0.1 ms; < 10 nm in diameter), constituting the raft precursors observed by others (for a review see [7]) to what is called resting state rafts, which are considered more stable and functional. We assume that protein clustering induces changes in the physical properties of the lipid bilayer of the raft domain making them different from the bulk membrane (see Fig. 10). The mechanism of these changes is still unknown, but we can speculate that clustering of cholesterol molecules in the presence of single flotillin dimers or other monomeric proteins form raft precursors. Clustering of flotillins may result in the recruitment of other typical raft lipids and proteins that would lead to the formation of resting-state rafts. This effect seems rather short-range, but it most probably includes both membrane leaflets. This hypothesis, although speculative to some extent, may help to understand the molecular mechanism(s) of how such proteins work.

The important question is whether EFR3A and MPP1 are the only resting-state raft domain organizing proteins. Considering flotillin-based resting state rafts alone, we might expect several other proteins to fulfill this role. Some of them could be tissue-specific, like MPP1, while others could be ubiquitous, such as EFR3A. This notion is based on the fact that both flotillin and EFR3A interactomes seem far from being completely explored.

The importance of the EFR3A’s apparent role as a resting state raft organizing protein could have implications for human health, in particular human cancer biology. Recent data revealed EFR3A interaction with KRAS, which was responsible for the nanoclustering of KRAS at the plasma membrane and hence activation of the MAPK signaling pathway. This interaction was considered important for tumorigenesis of pancreatic cancer, i.e., loss of EFR3A inhibited KRAS signaling and cancer progression [52]. Perhaps the nanoclustering of the latter at the plasma membrane observed by these authors is a result of the flotillin-EFR3A interaction described here.

## Conclusions

In this study, we demonstrated, for the first time, the interaction of flotillin-2 with the EFR3A protein. Employing various experimental approaches we confirmed that this interaction affects lateral membrane organization possibly playing a crucial role in resting state raft organization. This may implicate an important role in cell signaling. Therefore, it could have implications for human health, in particular human cancer biology.

### Supplementary Information

**Additional file 1.** Supplementary Figures and Table.

**Additional file 2.** Movie 1.

**Additional file 3.** Movie 2.

## Data Availability

The datasets generated during and/or analyzed during the current study are available from the corresponding author on reasonable request.

## Abbreviations

BCA: Bicinchoninic acid
BSA: Bovine serum albumin
BODIPY-SM: BODIPY™ FL C5-sphingomyelin
DMEM: Dulbecco’s modified Eagle’s medium (for cell culture growth)
DRM: Detergent-resistant membrane
DTT: Dithiothreitol
EDTA: Ethylenediaminetetraacetic acid
EFR3A: Eighty-five requiring 3A
EGF: Epidermal growth factor
EGFR: Epidermal growth factor receptor
Erk: Extracellular signal-regulated kinase
FAK: Focal adhesion kinase
FBS: Fetal bovine serum
FLIM: Fluorescence lifetime imaging microscopy
GAPDH: Glyceraldehyde-3-phosphate dehydrogenase
GPMVs: Giant plasma membrane vesicles
IPTG: Isopropyl β-d-1-thiogalactopyranoside
JNK: c-Jun N-terminal kinases
KnD: Knockdown
KRAS: GTPase KRas
LB: Luria–Bertani medium
MPP1: Palmitoylated membrane protein 1
MβCD: Methyl-β-cyclodextrin
MS: Mass spectrometry
pEGFR: Phosphorylated EGF receptor
PI3K: Phosphatidylinositol-3 kinase
PLCγ: Phospholipase C gamma
PMSF: Phenylmethylsulfonyl fluoride
PSD-95: Postsynaptic density protein 95
Ras: VHS: Vps27, Hrs, and STAM
Co-IP: Co-immunoprecipitation
SDS-PAGE: Sodium dodecyl sulfate polyacrylamide gel electrophoresis
shRNA: Short hairpin RNA
SPR: Surface plasmon resonance
Src: Sarcoma family kinases
svFCS: Spot variation fluorescence correlation spectroscopy
TBS-T: Tris-buffered saline with Tween 20
TCA: Tricholoroacetic acid
TMD: Transmembrane domain
