# Supplementary material for: EFR3A: a new raft domain organizing protein?
Source: Cell Mol Biol Lett. 2023 Oct 25;28:86. doi: 10.1186/s11658-023-00497-y (PMC10601247; doi:10.1186/s11658-023-00497-y)
Supplement: Supplementary file 1 — Additional file 1. Supplementary Figures and Table. [file 11658_2023_497_MOESM1_ESM.docx]

**Supplemental Data**

Title**: EFR3A: a new raft domain organizing protein?**

Magdalena Trybus*^1^, Anita Hryniewicz-Jankowska*^1^, Karolina Wójtowicz^2^, Tomasz Trombik^3^, Aleksander Czogalla^&1^, Aleksander F. Sikorski^&4^


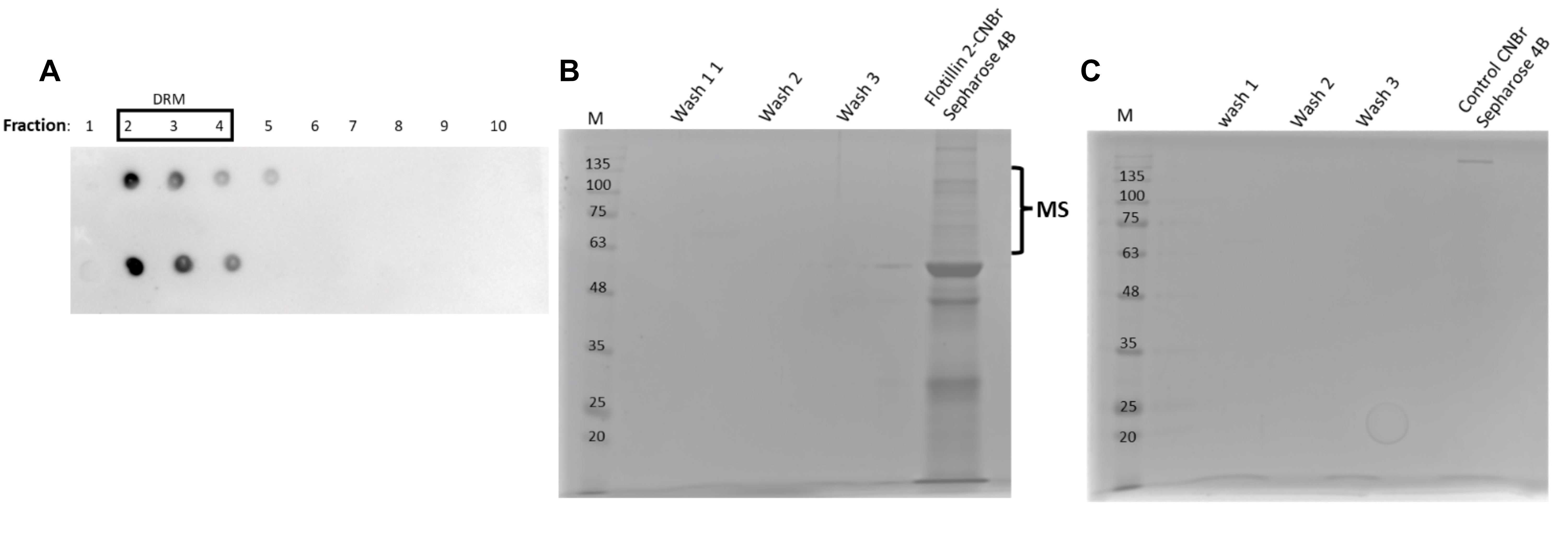


Figure S1. **Isolation of HeLa cells DRM fraction and “fishing out” flotillin partner protein(s) on flotillin-2 conjugated to CNBr-activated Sepharose 4B.** A. Isolation of DRM fraction and detection of GM1 via Cholera toxin B subunit conjugated to horseradish peroxidase. Separation of pull-down samples on SDS-PAGE gels stained with Coomassie Blue. B. Flotillin-2 bound resin. C. Control resin without flotillin-2. DRM fraction from HeLa cells diluted 1:1 with 2% octyl glucoside in 40 mM Tris-HCl, pH 7.4, 300 mM NaCl, and Protease Inhibitor Cocktail (Merck/Sigma-Aldrich) were incubated overnight with flotillin-2-Sepharose resin at 4°C. Then the resin was thoroughly washed with the same buffer, suspended in the sample buffer, and subjected to SDS-PAGE.

Table S1

Top 10 proteins identified as associated with membrane found in MS/MS analysis of proteins bound to immobilized on Sepharose 4B resin flotillin-2. As 11th E3 UFM1-protein ligase 1 is shown. Marked in yellow are proteins known to interact with flotillin (Liu W, et al. Oncol. Rep. 39, 45-52, 2018 and <https://string-db.org> ).

| Accession | Description | Mascott Score | Sequence Coverage (%) | Total Identified Peptides | Peptide RMS Mass Error (Da) | Total Identified fragments |
| --- | --- | --- | --- | --- | --- | --- |
| Q14156 | Protein EFR3 homolog A OS=Homo sapiens GN=EFR3A PE=1 SV=2 | 1661 | 30.7 | 23 | 0.002991219 | 277 |
| P21589 | 5~-nucleotidase OS=Homo sapiens GN=NT5E PE=1 SV=1 | 1470 | 26.8 | 13 | 0.003602777 | 168 |
| Q16891 | MICOS complex subunit MIC60 OS=Homo sapiens GN=IMMT PE=1 SV=1 | 1053 | 26.8 | 19 | 0.001890976 | 188 |
| P40939 | Trifunctional enzyme subunit alpha. mitochondrial OS=Homo sapiens GN=HADHA PE=1 SV=2 | 1032 | 25.8 | 15 | 0.003551807 | 196 |
| O95573 | Long-chain-fatty-acid--CoA ligase 3 OS=Homo sapiens GN=ACSL3 PE=1 SV=3 | 983 | 16.9 | 11 | 0.002889322 | 119 |
| P46459 | Vesicle-fusing ATPase OS=Homo sapiens GN=NSF PE=1 SV=3 | 679 | 22.7 | 13 | 0.006839478 | 150 |
| P28288 | ATP-binding cassette sub-family D member 3 OS=Homo sapiens GN=ABCD3 PE=1 SV=1 | 487 | 10.9 | 5 | 0.005387393 | 61 |
| Q9Y4F1 | FERM, RhoGEF and pleckstrin domain-containing protein 1 OS=Homo sapiens GN=FARP1 PE=1 SV=1 | 484 | 6.6 | 7 | 0.003651614 | 83 |
| Q14699 | Raftlin OS=Homo sapiens GN=RFTN1 PE=1 SV=4 | 350 | 10.4 | 4 | 0.002529328 | 54 |
| Q9C0B5 | Palmitoyltransferase ZDHHC5 OS=Homo sapiens GN=ZDHHC5 PE=1 SV=2 | 349 | 15 | 7 | 0.001795232 | 70 |
| O94874 | E3 UFM1-protein ligase 1 OS=Homo sapiens GN=UFL1 PE=1 SV=2 | 100 | 6.5 | 3 | 0.0065465 | 43 |


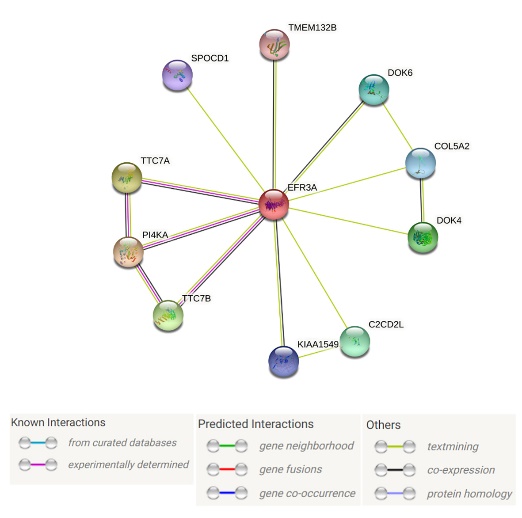


Figure S2. **Interaction of flotillin-2 with EFR3A protein has not been reported yet.** Data based on <https://string-db.org>


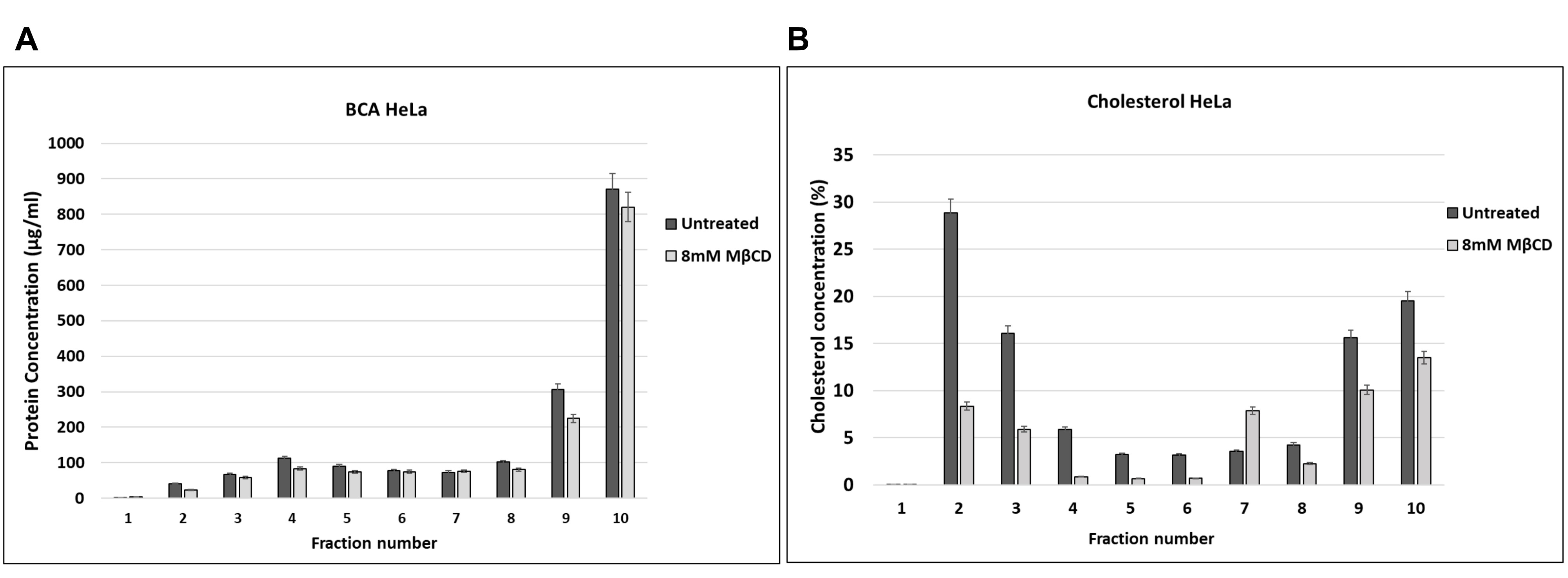


Figure S3. **Protein and cholesterol levels in DRM fractions.** Total protein (A) and cholesterol (B) content in the gradient fractions upon DRM isolation from cyclodextrin-treated and untreated HeLa cells.


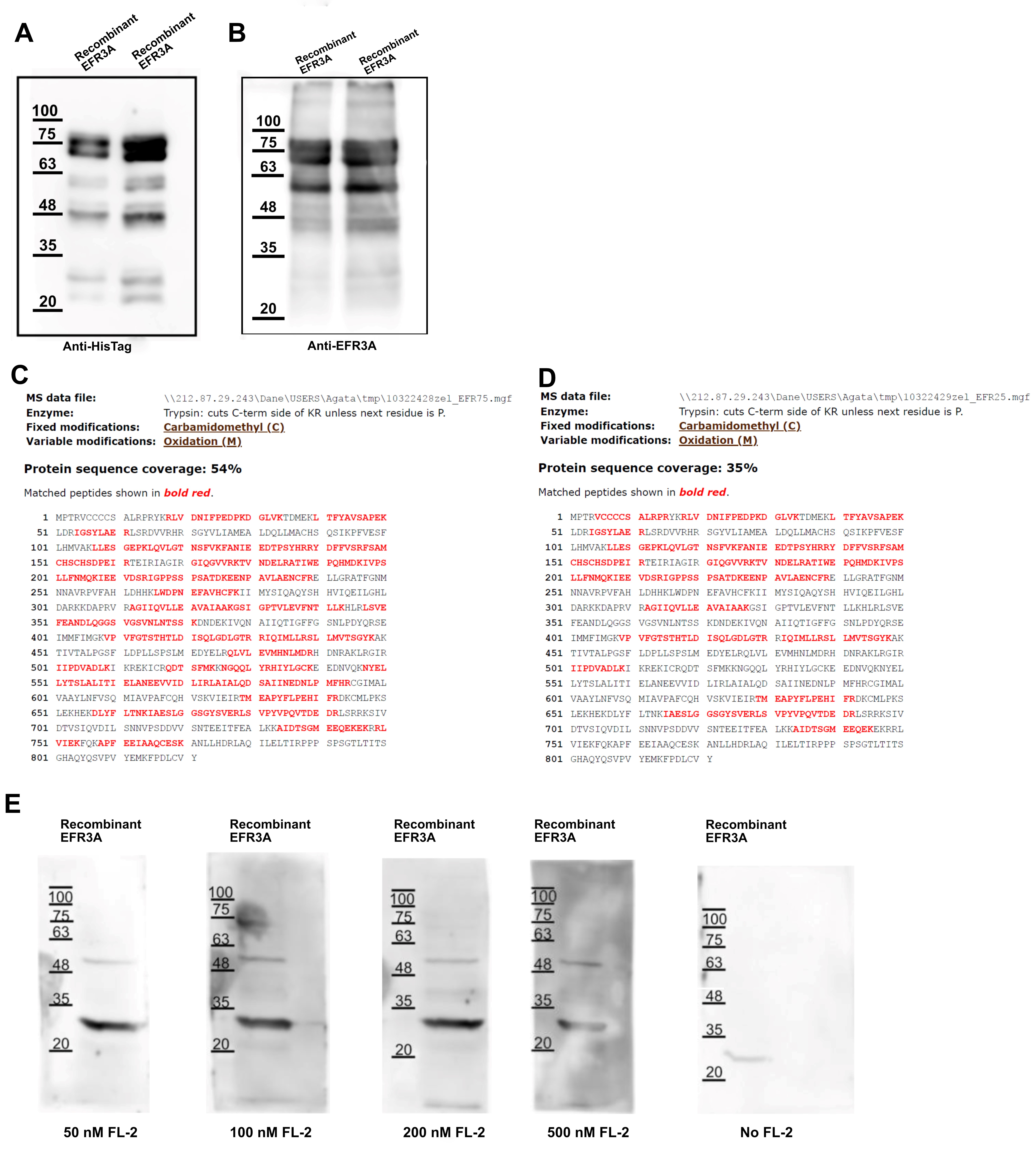


Figure S4. Bacterially expressed recombinant EFR3A and overlay assay. Recombinant EFR3A protein was purified on metal ion chelating resin (Ni2+) and subjected to SDS-PAGE, next the protein was subjected MS/MS identification and Western blotting. A) Western blotting with anti-HisTag antibodies and B) with anti-EFR3A antibodies. MS/MS identification: C) bands 75-96 kDa and D) 30 kDa. E) Overlay assay. Recombinant EFR3A protein was subjected to SDS-PAGE followed by transfer onto nitrocellulose. The membrane strips were incubated with increasing concentrations of recombinant flotillin-2, followed by incubation with goat anti-flotillin-2 antibodies (Abcam) and secondary donkey anti-goat antibodies (Santa Cruz).


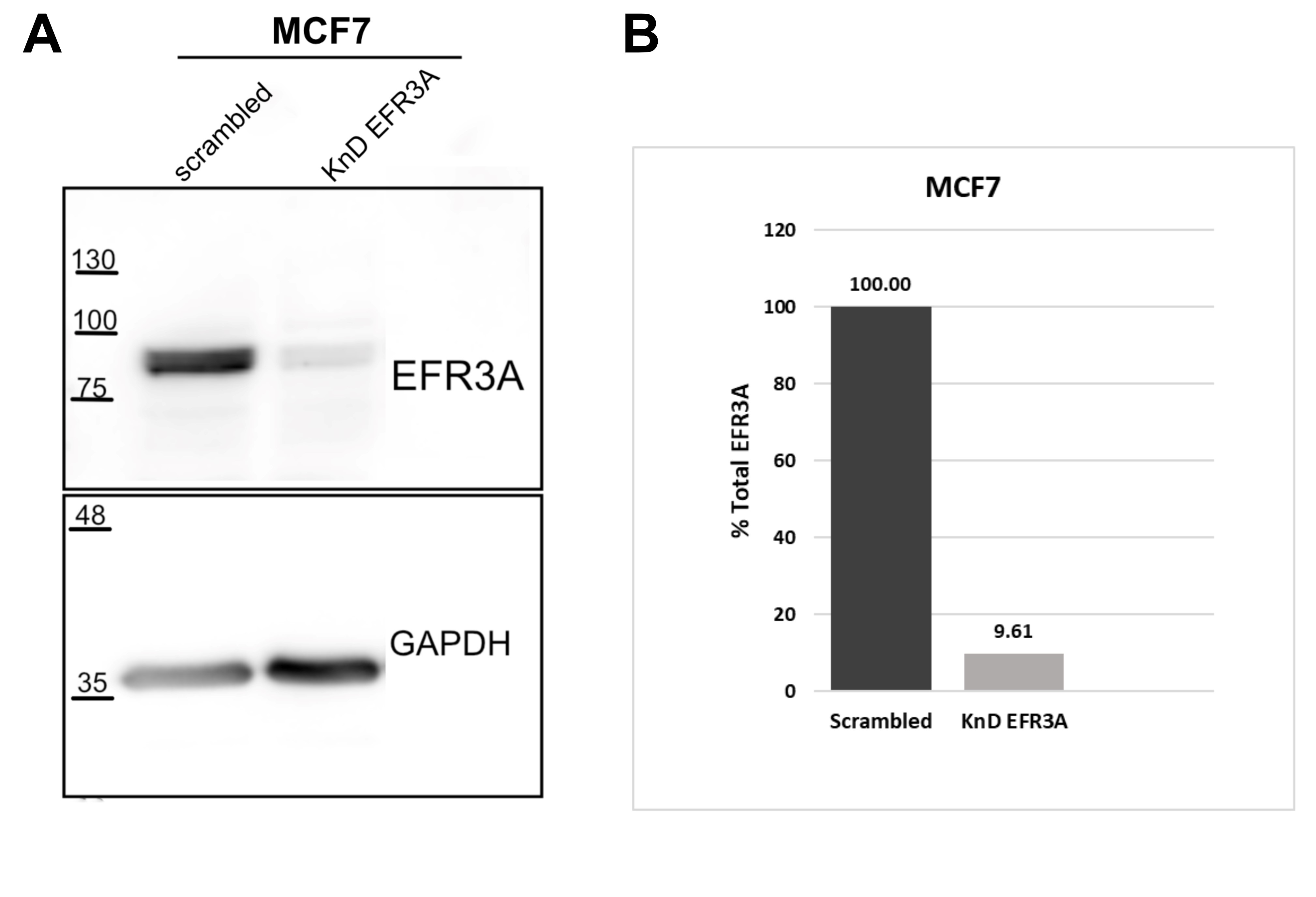


Figure S5. **Silencing expression of *EFR3A* gene in MCF7 cell line**. Stable cell lines were obtained using EFR3A shRNA Lentiviral Particles or “scrambled” shRNA Lentiviral Particles as described in the Methods section. A. Cell extracts of control or transduced cell lines were submitted to SDS PAGE and Western blotting was probed with anti-EFR3A antibodies. GAPDH visualization was used as a loading control. B. Quantitation of the EFR3A fractions showed in A.


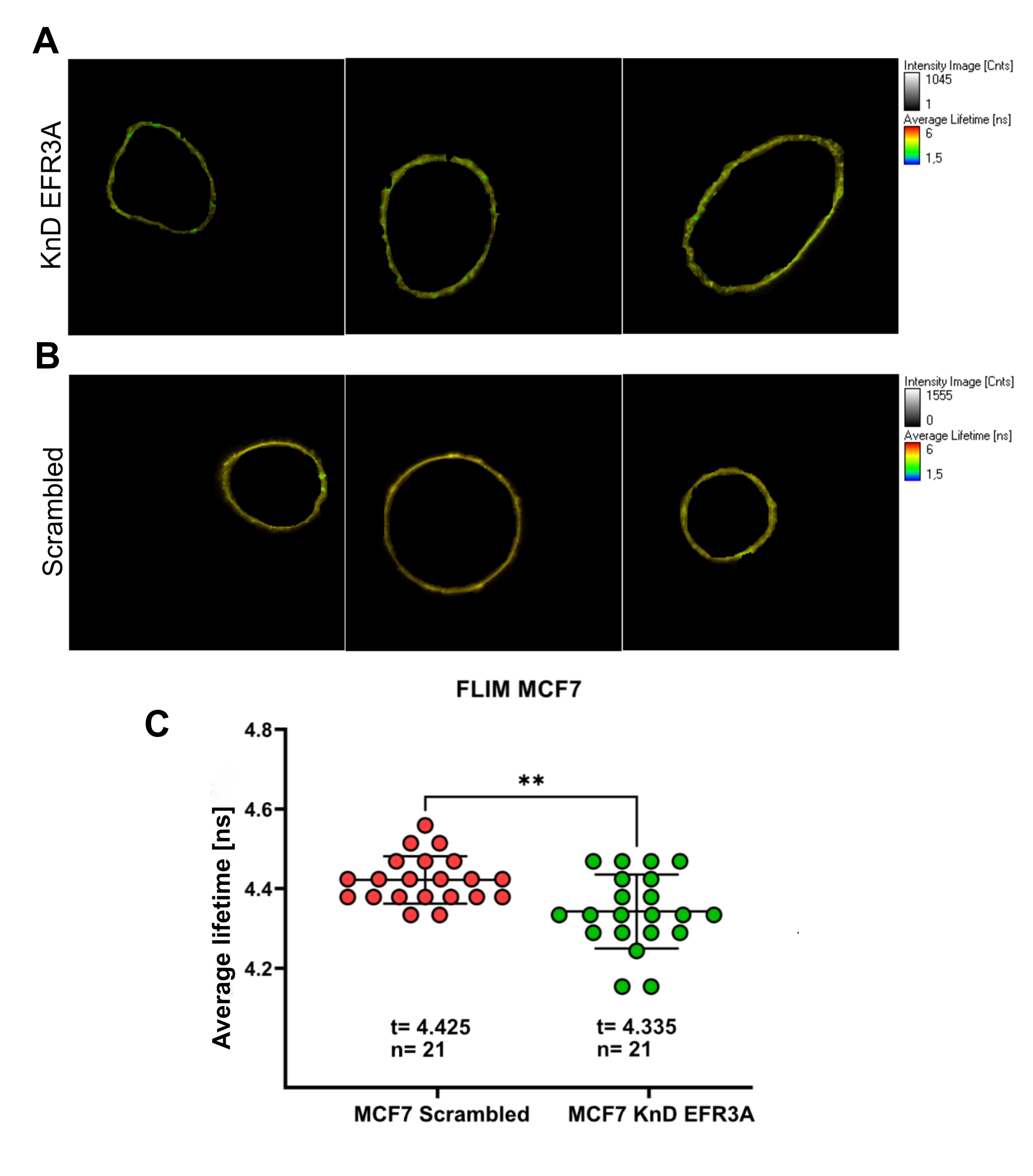


Figure S6. Examples of FLIM images of cell plasma membranes of A) scrambled MCF7, B) KnD *EFR3A,* and C) quantitation of FLIM data of *EFR3A* KnD and “scrambled” cells. For FLIM measurements of the fluorescence lifetime of the membrane-order sensitive probe, di-4 ANEPPDHQ (Invitrogen) control ”scrambled” and *EFR3A* KnD HeLa cells were grown on LabTek chambers in DMEM medium with 10% FBS. After 24h cells were washed twice and probed with 2 μM di-4, in DMEM medium for 5 min. Cells were washed twice in HBBS buffer with 10 mM HEPES pH 7.4. and measurements were performed.

MovieS1 KnD, and MovieS2 Scr1 **EGF-induced Ca^2+^ influx.** Example of recording of the real-time observations. Further details as in the legend to Fig. 9.
